# Alteration of BDNF and noradrenergic markers in locus coeruleus in a mouse model of cancer-induced bone pain

**DOI:** 10.1371/journal.pone.0330207

**Published:** 2025-08-14

**Authors:** Man Yuan, Long Zhang, Haili Zhu, Min Xie

**Affiliations:** School of Pharmacy, School of Basic Medical Sciences, Hubei University of Science and Technology, Xianning, China; Belgrade University Faculty of Medicine, SERBIA

## Abstract

The locus coeruleus (LC) is the principal source of noradrenaline (NA) in the central nervous system. In neuropathic pain states, nociceptive stimuli activate LC. This study examined the expression and localization of BDNF and NE neuron-specific proteins in the LC of mice with cancer-induced bone pain (CIBP). Behavioral experiments demonstrated that CIBP mice exhibited persistent spontaneous pain, mechanical and thermal hyperalgesia, and deficits in locomotor activity and motor coordination. H&E and TRAP staining revealed trabecular bone destruction and increased in osteoclast activity. Immunostaining showed elevated expression of neuronal activity marker c-Fos in the LC. Additionally, upregulation of noradrenergic markers - tyrosine hydroxylase (TH) and dopamine β-hydroxylase (DBH) – as well as brain-derived neurotrophic factor (BDNF), was observed in the LC. In vitro studies indicated that inhibition of the BDNF/TrkB signalling pathway reduced the expression of noradrenergic markers. Anterograde tracing, achieved by Fluoro-ruby injection into the LC and subsequent detection of Fluoro-ruby colocalised with TH and DBH in spinal cord, confirmed LC-spinal cord projections. Immunofluorescence analysis demonstrated increased fluorescence intensities of TH, DBH, c-Fos, phosphorylated cAMP-response element- binding protein (pCREB), and α_2A_ receptor in the spinal cord, alongside reduced intensities of enkephalin (ENK) and GABA A Receptor β2 (GABRB2). Western blotting further corroborated elevated expression levels of TH and c-Fos in spinal cord tissue. In summary, CIBP mice exhibited enhanced neuronal activity in the LC, upregulation of noradrenergic markers, and BDNF/TrkB-mediated modulation of noradrenergic neurons. Concurrently, inhibitory signalling was attenuated in the SDH.

## Introduction

Breast cancer is the most prevalent malignancy among women worldwide, with approximately 2.2 million new cases reported in 2020, accounting for 11.7% of all cancer diagnoses [1]. Patients with breast cancer often develop distant metastases, most commonly occurring in the bone, liver, and lungs [2]. Bone metastases from breast cancer lead to osteolytic destruction, resulting in severe complications such as bone pain, pathological fracture, and paralysis due to spinal cord compression[3]. The presence of metastatic tumor cells in bone not only drives bone degradation but also directly contributes to pain generation [4]. Furthermore, bone metastasis markedly increases the risk of painful skeletal-related events (SREs), with reported incidence rates ranging from 17% to 60% [5]. Given the high prevalence of pain and SREs, effective pain management – whether through pharmacologic interventions or radiotherapy – remains a critical component of metastatic breast cancer treatment to improve patients’ quality of life [6].

Injury to bone-innervating neurons is one of the mechanisms underlying bone metastasis-induced pain [7]. Cancer cells growing within bone damage the sensory fibers that innervate the adjacent bone tissue [8]. In response to local nociceptive stimuli, the peripheral sensory nerve fibers became highly sensitised [9]. Upon excitation, the primary afferent fibers release excitatory neurotransmitters in the spinal dorsal horn (SDH), leading to the sensitization of nociceptive pathways [10].Within these pathways, the brainstem – including the periaqueductal grey (PAG), rostral ventromedial medulla (RVM), and locus coeruleus (LC) – plays a crucial role in nociception and pain processing [11]. Neuroimaging studies have demonstrated enhanced functional connectivity between brainstem pain-modulatory regions (RVM, PAG and LC) under neuropathic pain condition [12]. Furthermore, electrophysiological alterations have been observed in neurons of the spinal cord and brainstem pain-modulatory regions in cancer-induced pain models [13, 14].

The locus coeruleus (LC) is a dense cluster of noradrenaline (NA)-producing neurons located deep within the brainstem and serves as the primary source of noradrenergic projections throughout the central nervous system [15]. The LC-NA system plays a critical role in regulating diverse physiological and behavioural processes, including arousal, cognition, memory and sensory perception [16]. Notably, this system also significantly contributes to the modulation of both acute and chronic pain [17]. The LC contributes its analgesic effects chiefly through descending projections to the spinal cord, where it modulates nociceptive transmission in the superficial dorsal horn [18]. In response to noxious stimuli, LC neurons become activated and influence the ascending pain pathway – a phenomenon well-documented in neuropathic pain models [19]. Correspondingly, nerve injury induces increased expression of phosphorylated cAMP-response element-binding protein (pCREB) and the immediate-early gene c-Fos in LC, indicative of heightened neuronal excitability [20]. Peripheral nociceptive signals are relayed to the brain via spinal projection neurons, which constitute key elements of the ascending pain pathway [21]. In mouse models of cancer‑induced bone pain (CIBP), glial cells activation and upregulation of the NLRP3 inflammasome signalling pathway have been observed in the spinal dorsal horn, further promoting central sensitisation [22]. Collectively, these findings suggest that targeting the LC may provide foundation for developing novel analgesic strategies.

Brain derived neurotrophic factor (BDNF) promotes neuronal growth and modulates synaptogenesis and synaptic plasticity [23]. Within the central nervous system, BDNF serve as a potential biomarker for neuropathic pain and functions across multiple pain-processing regions, including the dorsal root ganglion, spinal cord, and supraspinal areas [24]. BDNF exerts its biological effects primarily through tropomyosin-related kinase B (TrkB) receptor, with spinal BDNF/TrkB signaling being implicated in neuropathic pain mechanism [25]. The BDNF/TrkB pathway contributes to central sensitisation, manifesting as allodynia, hyperalgesia, and spontaneous pain by enhancing the excitability of second-order neurons in the spinal dorsal horn [26]. In the LC, TrkB receptor agonist treatment reduces painful stimulation-induced GABA release, activates noradrenergic neurons, and promotes noradrenaline releases in the spinal dorsal horn [27]. To further elucidate BDNF’s regulatory effects on the noradrenergic system under pathological pain conditions, we employed both *in vivo* and *in vitro* approaches. *In vivo*, a cancer-induced bone pain (CIBP) mouse model was used to assess behavioural, cellular, and molecular changes in LC. Given the LC’s size small – comprising only approximately 3,000 brain cells [16] – isolating pure LC neurons for cellular studies is challenging. Therefore, the Cath.a cell line, a locus coeruleus-like noradrenergic model, was utilised for in vitro experiments. Derived from a brainstem tumour in transgenic mice expressing the SV40 large T antigen under the tyrosine hydroxylase promoter, this cell line exhibits key features of noradrenergic neurons, including synaptophysin expression and high levels of noradrenaline and dopamine [28, 29]. We treated Cath.a cells with the TrkB receptor antagonist ANA-12 to investigate BDNF/TrkB signalling’s influence on noradrenergic markers. By combining in vivo and in vitro models, this study provides insights into BDNF’ role in noradrenergic neurons under chronic pain conditions.

In this study, we investigated alterations in noradrenergic markers and BDNF within the LC in CIBP. To achieve this, we established a CIBP mouse model, examined the expression and localization of specific proteins for noradrenergic neurons in the LC and assessed the changes in noradrenergic markers in Cath.a cells following TrkB antagonist treatment.

## Materials and methods

### Animals

A total of 27 male C57BL/6 mice (weighing 18–20 g) were obtained from the Hubei Provincial Experimental Animal Center (Wuhan, China). The animals were housed in a temperature-controlled facility (22 ± 1°C) under a 12/12 h light-dark cycle with ad libitum access to food and water. Every effort was made to minimise the number of animals used and to reduce their discomfort. All experimental procedures were approved by the Ethics Committee for Laboratory Animals at Hubei University of Science and Technology (Approval No. 2022-04-050).

### Cell culture and treatment

The mouse breast carcinoma cells (4T1 cells) were cultured in RPMI‑1640 medium supplemented with 10% fetal bovine serum, 50 U/ml penicillin and 50 μg/ml streptomycin at 37°C in a humidified atmosphere containing 5% CO₂.

Cath.a cells were maintained in complete Cath.a cell medium under the same conditions (37 °C, 5% CO₂). The experiment consisted of four groups: Control, cells were cultured without additional treatment. BDNF-treated (BDNF), cells were stimulated with BDNF at a final concentration of 1 μg/mL for 24 hours. ANA-12-treated (ANA-12), cells were treated with ANA-12 alone at a final concentration of 1 μM for 24 hours in the absence of BDNF. BDNF+ANA-12 co-treated (BDNF+ANA-12), cells were first pretreated with 1 μg/mL BDNF for 24 hours, followed by co-incubation with 1 μM ANA-12 for an additional 24 hours under identical culture conditions. All experiments were performed in biological replicates under consistent culture conditions.

### Establishment of the mouse model of CIBP

Mice were acclimated to the environment for one week prior to the experiment. They randomly allocated into two groups: the sham group and CIBP group, with nine mice per group. Bone pain was induced using 4T1 cells. Briefly, the animals were anaesthetized via intraperitoneal injection of pentobarbital sodium (50 mg/kg), and the left hind limb was shaved and disinfected with 70% ethanol. A hole was then drilled into the left tibia, a microsyringe containing 3.5 × 10^5^ cancer cells in HBSS was slowly injected into the intramedullary space. Sham-operated mice received an equivalent volume of vehicle (normal saline).

### Behavioural analysis

Behavioural assessments were conducted on days 0, 4, 7, and 14 post-modelling to evaluate nociceptive behaviour changes in each group. The testing protocol comprised: Paw withdrawal threshold (PWT) measurement, spontaneous flinches assessment, hot plate test, rotarod performance evaluation.

Paw withdrawal threshold (PWT): Mice were individually placed in transparent glass chambers (30 × 30 × 30 cm) and allowed to acclimatise for ≥ 30 min prior to testing. Mechanical sensitivity was evaluated on the left hind paw using von Frey filaments (Stoelting, Wood Dale, IL, USA; range: 0.008–6.0 g). Filaments were applied perpendicular to the plantar surface with sufficient force to cause slight bending, maintained for 3–5 s. A positive response was recorded upon paw withdrawal or lifting; absence of movement constituted a negative response. Following a positive response, the next lower-force filament was applied; after a negative response, the subsequent higher-force filament was used. Each mouse received six filament applications. The 50% paw withdrawal threshold was calculated using the formula: 50% PWT (g) = 10^[Xf + kδ]/ 10,000, where *Xf* = value (log units) of the final filament used, *k* = Dixon’s up-down method constant, and *δ* = mean difference (log units) between stimuli.

Spontaneous flinch test: Mice were individually housed in transparent glass chambers (30 × 30 × 30 cm) with a 30-minute acclimatisation period prior to testing. Spontaneous nociceptive behaviour was quantified by counting paw withdrawal events during three consecutive 5-minute observation sessions.

Rotarod test: The accelerating rotarod test was employed to assess motor coordination and balance [30]. Briefly, mice were acclimatised to the rotarod apparatus (Beijing Zhongshi Dichuang, China) being placed on the rotating rod. Prior to the formal test, the mice underwent training sessions consisting of three trials per day at a constant speed of 4 rpm for 10 min, conducted over three consecutive days. Trials were separated by 10-minute intervals. During the test, the initial speed was set to 10 rpm for 10 s, followed by a 10-second acceleration phase. The speed then increased to 20 rpm and was maintained for 30 s before another acceleration period. Each session lasted 10 min, and every mouse performed three trials with 10-minute rest intervals between them. The latency to fall from the rod and the total distance travelled were recorded.

Hot plate test: The hot plate test was employed to evaluate thermal nociception. Mice were placed individually on a heated plate (Techman, China) maintained at 55°C. The paw withdrawal latency (PWL) was recorded as the time taken for the mouse to exhibit a nociceptive response (paw lifting, licking, or jumping). To prevent tissue injury, a cut-off time of 30 s was imposed [31]. Each hind paw was tested three times, with left and right paws alternated between trials. A minimum interval of 5 min was maintained between successive tests to minimise residual effects. The average PWL across trials was calculated for statistical analysis.

### Histological analysis of bone structure

On day 14 post-surgery following behavioural tests, mice were euthanized with an overdose of pentobarbital sodium (150 mg/kg). The left tibias were collected, fixed in 4% paraformaldehyde for 24 h, and decalcified in 10% EDTA solution. Following decalcification, the bone tissue was dehydrated, embedded in paraffin, and sectioned into 4 μm slices. For hematoxylin and eosin (H&E) staining, sections were stained with hematoxylin solution for 3 min, rinsed briefly in tap water (10 s), counterstained with eosin for 3 min, and washed again in tap water (10 s). Dehydration and clearing were performed sequentially through: 75% ethanol (10 s), 85% ethanol (10 s), 95% ethanol (10 s), absolute ethanol (30 s, twice), and xylene (1 min, twice). For tartrate-resistant acid phosphatase (TRAP) staining, sections were first treated with xylene (10 min, twice), followed by graded ethanol series: absolute ethanol (5 min), 90% ethanol (2 min), and 70% ethanol (2 min), then rinsed in distilled water (2 min). Sections were subsequently incubated in substrate solution (protected from light, 60 min), rinsed in water (1 min), counterstained with methyl green (2–3 min), dehydrated and cleared, and finally mounted with neutral gum.

### Stereotaxic injections

Anterograde tracing from the LC to the spinal cord was performed using Fluoro-ruby injections into the LC. Briefly, 6–8-week-old C57BL/6 mice were anesthetised by sodium pentobarbital and secured in a stereotaxic apparatus. For LC injections, the scalp was disinfected, the skin incised, and mucosal tissue on the skull surface removed. The skull was aligned anterior-posteriorly and mediolaterally using the Bregma and Lambda reference points. The injection site of the locus coeruleus (AP = −5.3 mm, ML = −0.8 mm, DV = −4.0 mm) were determined according to a mouse brain stereotaxic atlas. A small burr hole was drilled, and 100 nl of Fluoro-ruby were injected via a microsyringe, followed by a 5–10 min dwell time to minimise backflow.

### Immunohistochemistry

Following behavioral testing, the mice were deeply anaesthetised and transcardially perfused with 4% paraformaldehyde. The brainstem and spinal cord tissues were dissected and embedded in paraffin. Sections were cut at a thickness of 4 μm and subsequently deparaffinized. Antigen retrieval was performed by immersing the sections in antigen retrieval buffer and heating at 100°C for 15 min. Endogenous peroxidase activity was quenched by incubating the sections in 3% hydrogen peroxide for 10 min, followed by three 5-min washes in PBS. After carefully removing excess moisture around the tissue sections using filter paper, a hydrophobic barrier pen was used to encircle the tissue, which was then covered with blocking solution for 60 min at room temperature. The sections were incubated overnight at 4°C with the following primary antibodies: anti-tyrosine hydroxylase (A25683, rabbit, 1:200; ABclonal), DBH (10777–1-AP, rabbit, 1:200; Proteintech), and anti-c-Fos (AF5354, rabbit, 1:100; Affinity). After primary antibody incubation, the sections were washed three times in PBS (5 min per wash). A secondary antibody incubation was performed at room temperature for 1 h. The sections were then treated with 3,3′-diaminobenzidine (DAB) chromogenic reagent, with development monitored under a microscope (2–30 min), followed by rinsing in double-distilled water (ddH₂O). Counterstaining was conducted using haematoxylin for 30 min, followed by ddH₂O rinses. Dehydration was performed sequentially in 85% ethanol (10 s), 95% ethanol (10 s), absolute ethanol I (30 s), and absolute ethanol II (30 s), before clearing in xylene (two rounds, 1 min each). Finally, coverslips were mounted using neutral resin. Images were acquired using an IX73 fluorescence microscope (Olympus, Japan). For each marker (TH, DBH, and c-Fos), three sections containing the locus coeruleus were selected for manual quantification of positively stained cells. To ensure objectivity and minimise bias, cell counting was performed by an investigator blinded to the experimental groups.

### Immunofluorescence

Paraffin-embedded tissue sections were deparaffinised in xylene and underwent antigen retrieval. Endogenous peroxidase activity was blocked by incubating sections in 3% hydrogen peroxide for 10 min. Primary antibody incubation was performed at 4°C for 24 h using the following antibodies: BDNF (DF6387, rabbit, 1:100; Affinity), phospho-CREB (AF3189, rabbit, 1:100; Affinity), c-Fos (AF5354, rabbit, 1:100; Affinity), tyrosine hydroxylase (A25683, rabbit, 1:200; ABclonal), DBH (10777–1-AP, rabbit, 1:100; Proteintech), adrenergic receptor α-2A (DF3076, rabbit, 1:100; Affinity), NeuN (66836–1-Ig, mouse, 1:100; Proteintech), GABRB2 (A1876, rabbit, 1:100; ABclonal), PENK (A6302, rabbit, 1:100; ABclonal), and tyrosine hydroxylase (#45648, mouse, 1:200; Cell Signaling Technology). Following three PBS washes, sections were incubated for 1 h at room temperature with appropriate secondary antibodies: goat anti-rabbit IgG H&L (FITC) (ab6717, 1:1000; Abcam) and goat anti-mouse IgG H&L (TRITC) (ab6786,1:1000; Abcam). Nuclear counterstaining was conducted using Hoechst 33342 (C1026; Beyotime, China) for 15 min. Sections were mounted with antifade mounting medium suitable for immunofluorescence. Imaging was performed using an IX73 fluorescence microscope (Olympus, Japan), with fluorescence intensity quantified using ImageJ software.

### Western blotting

Spinal cord tissues were collected and homogenized on ice in RIPA lysis buffer supplemented with 1% protease inhibitor cocktail. Following centrifugation at 12,000 × g for 20 min at 4°C, the supernatant was collected. An appropriate volume of 4 × loading buffer was added to the supernatant, mixed thoroughly, and samples were denatured at 100 °C for 10 min using a metal heating block. Protein concentrations were determined using a BCA assay. Equal protein quantities were separated by SDS-PAGE and transferred onto polyvinylidene fluoride (PVDF) membranes. After blocking with 5% non-fat milk for 1.5 h at room temperature, membranes were incubated overnight at 4°C with primary antibodies targeting c-Fos (AF5354, rabbit, 1:1000; Affinity), tyrosine hydroxylase (A25683, rabbit, 1:1000; ABclonal) and β-actin (AF7018, Rabbit,1:1000; Affinity). Following three washes with Tris-buffered saline with Tween-20 (TBST), membranes were incubated for 1 h at room temperature with HRP-conjugated goat anti-rabbit IgG (H + L) HRP secondary antibody (AS014, 1:10000; ABclonal). Protein bands were visualised using enhanced chemiluminescence detection, and band intensities were quantified using ImageJ software.

Following treatment, Cath.a cells were harvested into centrifuge tubes and centrifuged to remove the culture medium. The cell pellets were resuspended in pre-chilled PBS, centrifuged again, and the supernatant was aspirated. The cells were then kept on ice. Lysis was performed using lysis buffer supplemented with 1% protease inhibitor cocktail, and the mixture was incubated on ice for 20 min to ensure complete lysis. Subsequently, 4 × loading buffer was added to the lysates, and the samples were heated in a boiling water bath for 10 min to denature the proteins. The lysates were then sonicated for approximately 15 sec and centrifuged to collect the supernatants. Western blotting was performed as described for the spinal cord tissue samples.

### Statistical analysis

All statistical analyses were performed using SPSS 25. Normally distributed continuous data were compared between two groups using an independent sample t-test. Correlations were assessed using Spearman’s rank correlation analysis. Other data were analysed by one-way analysis of variance followed by Tukey’s post-hoc test. Behavioral test data are expressed as mean ± SEM. Immunofluorescence, immunohistochemical, and Western blot data are presented as mean ± SD. A p-value <0.05 was considered statistically significant.

## Results

### Pain-related behaviors are induced in a mouse model of CIBP

**Fig 1:**
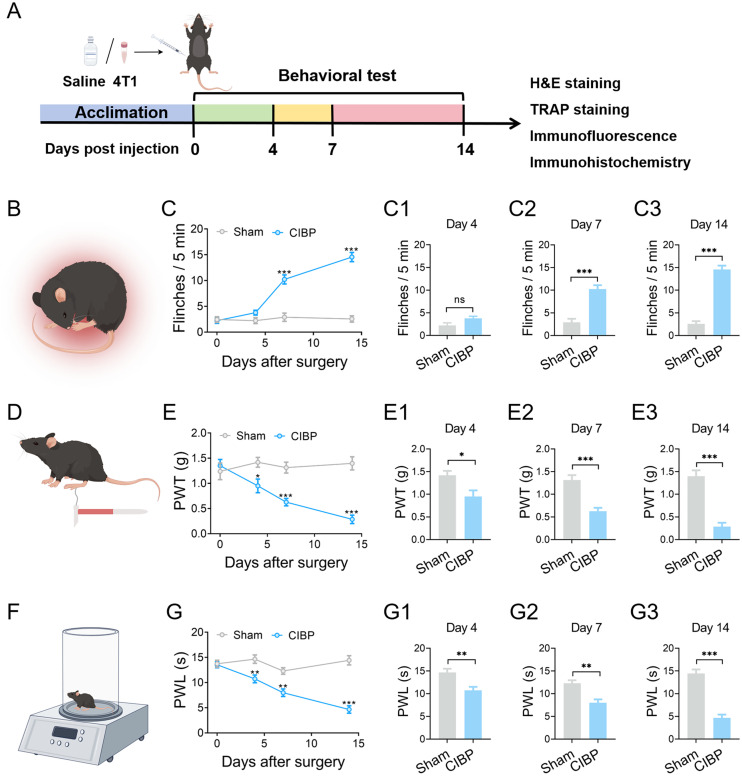
Pain-related behaviours changes in sham and CIBP mice. (A) Schematic of the experimental timeline. Behavioural tests – including spontaneous flinching, PWT, and PWL – were conducted on day 0, 4, 7, and 14 post-surgery. (B, D, F) Schematics illustrating of spontaneous flinches, PWT and PWL test paradigms. (C, E, G) Statistical analysis of the numbers of spontaneous flinches, PWT response to von Frey filaments and PWL responses to thermal stimuli. Data are presented as mean ± SEM (n = 9). *P < 0.05, **P < 0.01, and ***P < 0.001 vs. sham mice. Abbreviations: CIBP, cancer‑induced bone pain; PWT, paw withdrawal threshold; PWL, paw withdrawal latency.

A mouse model of CIBP was established by the inoculation of 4T1 cells into the tibial bone marrow cavity. Pain-related behaviours were assessed post-operatively following the experimental protocol (Fig 1A). Intratibial inoculation of 4T1 cells elicited significant pain-related behaviours, manifested as increased spontaneous pain responses and enhanced, mechanical, and thermal hypersensitivity. Compared with sham mice, CIBP mice exhibited a significant increase in the numbers of spontaneous flinches, with values rising to 3.78 ± 0.46 on day 4 (*P* > 0.05 vs. sham group), 10.22 ± 0.88 on day 7 (*P* < 0.05 vs. sham group), and 14.56 ± 0.88 on day 14 (*P* < 0.05 vs. sham group; Fig 1C); Similarly, the PWT of CIBP mice decreased to 0.95 ± 0.13 g on day 4 (*P* < 0.05 vs. sham group), 0.63 ± 0.07 g on day 7 (*P* < 0.05 vs. sham group), and 0.29 ± 0.08 g on day 14 (*P* < 0.05 vs. sham group; Fig 1E); Furthermore, the PWL in response to thermal stimuli was reduced in CIBP mice, declining to 10.73 ± 0.77 s on day 4 (*P *< 0.05 vs. sham group), 8.00 ± 0.75 s on day 7 (*P* < 0.05 vs. sham group), and 4.67 ± 0.73 s on day 14 (*P* < 0.05 vs. sham group; Fig 1G). These results indicate that cancer cell implantation induced hyperalgesia.

### Motor coordination, balance and bone structure are impaired in CIBP mice

**Fig 2:**
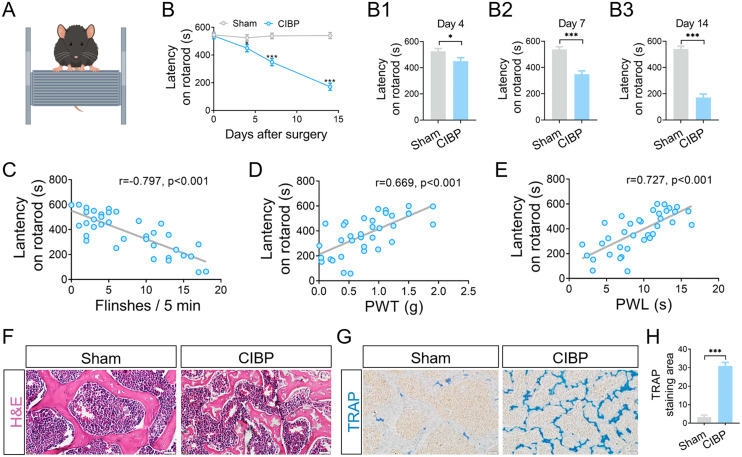
Motor coordination and bone structure changes in sham and CIBP mice. (A) Schematic of the rotarod test, in which mice were placed on an accelerating rotating rod. (B) Statistical analysis of latency to fall in the rotarod test. Data are presented as mean ± SEM (n = 9). (C-E) Linear regression analysis of motor coordination with spontaneous flinches, PWT, and PWL. (F, G) Representative images of H&E and TRAP staining in tibial sections (n = 3). Scale bar = 20 μm. (H) Quantitative analysis of the TRAP-positive area. *P < 0.05, **P < 0.01, ***P < 0.001 vs. sham mice. Data are presented as mean ± SD. Abbreviations: H&E, hematoxylin and eosin; TRAP, tartrate-resistant acid phosphatase.

The mice were tested on a rotarod were apparatus to assess motor function. CIBP mice demonstrated significantly impaired locomotor activity and motor coordination compared to sham mice. Quantitative analysis revealed that the latency to fall in CIBP mice was markedly reduced to 449.97 ± 26.57 s on day 4 (*P* < 0.05 vs. sham group), 348.57 ± 25.78 s on day 7 (*P* < 0.05 vs. sham group), and 171.41 ± 25.83 s on day 14 (*P* < 0.05 vs. sham group; Fig 2B). Correlation analysis between fall latency and pain behaviors (Fig 2C-2E) suggested that the decreased rotarod performance in CIBP mice was likely attributable to increased pain sensitivity. Following behavioral testing, bone tissues samples were collected for histological examination. H&E staining revealed substantial trabecular bone loss in CIBP mice at 14 days post-surgery, whereas sham-operated mice showed no apparent bone destruction (Fig 2F). TRAP staining demonstrated significantly enhanced osteoclast activity in CIBP mice compared to sham mice (*P* < 0.05; Fig 2G-2H), consistent with the bone degradation observed in H&E-stained sections.

### Detection of noradrenergic markers and neuron activity marker in LC neurons

**Fig 3:**
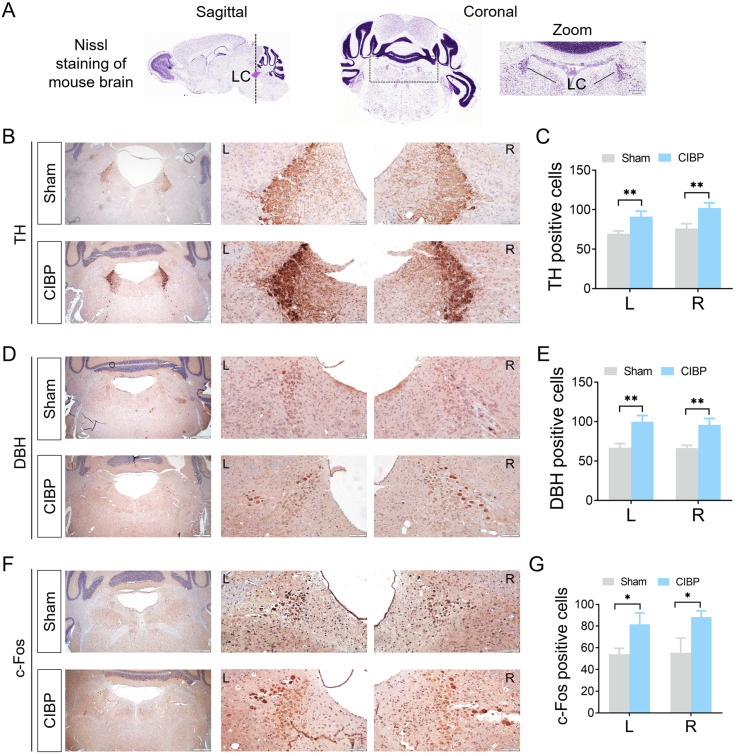
Characterization of LC neuronal markers. (A) Localization of the LC in the brain (Allen Brain Atlas). Scale bar = 262 μm. (B, D) Representative images immunohistochemical TH and DBH expression in the LC. Scale bar: overview (first lane) = 500 μm; higher magnification (second and third panels) = 100 μm. (C, E) Quantitative analysis of TH- or DBH-positive cells in the LC (n = 3). (F, G) Quantitative analysis of c-Fos-positive cells in the LC (n = 3). *P < 0.05, **P < 0.01 vs. sham mice. Data were presented as mean ± SD. Abbreviations: LC, locus coeruleus; TH, tyrosine hydroxylase; DBH, dopamine β hydroxylase.

Noradrenergic neurons in the LC were identified through immunohistochemical staining of TH and DBH, two key enzymes in norepinephrine biosynthesis [32]. The anatomical position of the LC in the mouse brain was verified using Nissl staining data from Allen Brain Atlas (https://mouse.brain-map.org/experiment/thumbnails/100048576?image_type=atlas), as shown in Fig 3A. According to the Allen Brain Atlas, we detected the localization of the two noradrenergic markers, tyrosine hydroxylase (TH) and dopamine β-hydroxylase (DBH). Immunohistochemical analysis of TH and DBH in LC is shown in Fig 3B and 3D. Compared with sham-operated mice, the number of TH- and DBH-labeled LC neurons was significantly increased bilaterally in CIBP mice (*P *< 0.05 vs. sham group; Fig 3C and 3E). Additionally, the neuronal activity marker c-Fos was examined in the LC. As shown in Fig 3F and 3G, the c-Fos-labelled LC neurons were significantly increased in CIBP mice (*P* < 0.05 vs. sham group). These results suggest the activation of noradrenergic neurons in the LC.

### The regulatory effect of BDNF on noradrenergic neurons

**Fig 4:**
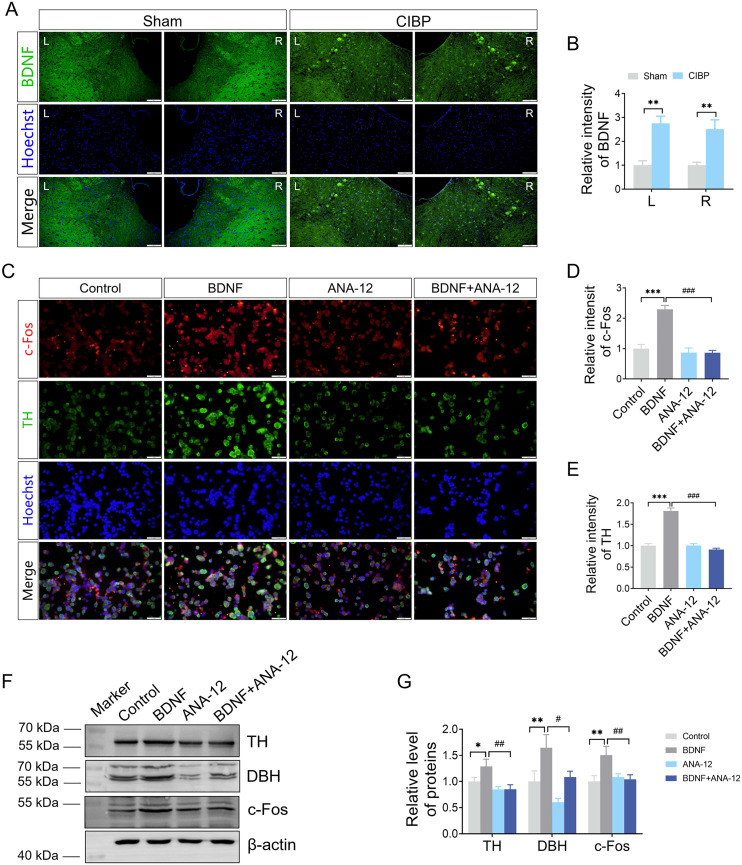
BDNF expression in the LC and its effect on noradrenergic marker. (A) Representative immunofluorescence images of BDNF in the LC. (B) Quantitative analysis of BDNF fluorescence intensity (n = 3). (C) Colocalization of c-Fos (red) and TH (green) in Cath.a cells, counterstained with Hoechst 33342 (blue). Scale bar = 20 μm. (D, E) Quantitative analysis of c-Fos and TH fluorescence intensities in Cath.a cells following BDNF, ANA-12, and combination treatments. Data are presented as mean ± SD (n = 3). (F) Western blot analysis of TH, DBH and c-Fos expression in Cath.a cells. (G) Quantitative analysis of TH, DBH and c-Fos protein level (n = 3). *P < 0.05, **P < 0.01 vs. control group. ^**#**^*P* < 0.05, ^**##**^*P* < 0.01, ^**###**^*P* < 0.001 vs. BDNF group. Data were expressed as mean ± SD.

BDNF is a crucial neuromodulator in pain transmission and an important regulator of noradrenergic neuron function [33, 34]. We subsequently detected the BDNF expression in the LC and investigated the effect of the BDNF/TrkB signalling pathway on the expression of noradrenergic markers in Cath.a cells, which express TH and DBH. The increased fluorescence intensity of BDNF was observed in LC of CIBP mice (Fig 4A-4B). In cellular studies, BDNF treatment elevated in c-Fos and TH expression levels, whereas these increases were attenuated by the TrkB antagonist ANA-12 (Fig 4C-4E). Immunoblot analysis further confirmed that ANA-12 treatment reduced the protein levels of TH, DBH, and c-Fos in BDNF- stimulated cells (Fig 4F-4G). These results demonstrate that BDNF regulates noradrenergic marker expression and neuronal activity.

### Changes of neuron activity markers in LC and SDH

**Fig 5:**
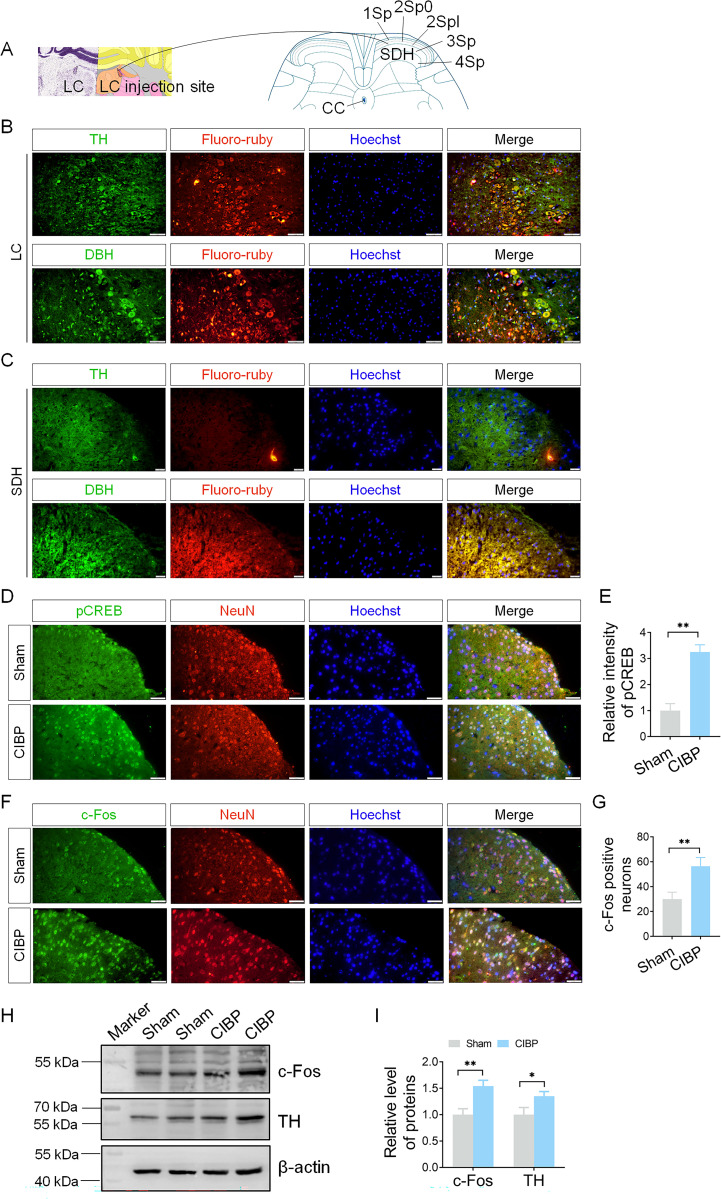
Neuronal tracing from the LC to the spinal cord. (A) Schematic of LC-spinal cord projections (Allen Brain Atlas), indicating the LC injection site. (B, C) Fluoro-ruby was injected into the LC, with colocalization of Fluoro-ruby and TH/DBH observed in both LC and SDH. Scale bars = 50 μm (B), 20 μm (C). (D, F) Colocalization of p-CREB/c-Fos (green) with the neuronal marker NeuN (red) in SDH. Scale bar = 20 μm. (E) Quantitative analysis of fluorescence intensity in (D) (n = 3). (G) Quantitative of c-Fos-positive neurons in SDH (n = 3). (H) Western blot analysis of c-Fos and TH expression in spinal cord tissue. (I) Quantitative analysis of c-Fos and TH protein level (n = 3). *P < 0.05, **P < 0.01 vs. sham group. Data were expressed as mean ± SD.

Since projections from the LC to the SDH can regulate ascending nociceptive information [18], we next examined changes in neuronal activity within the SDH. First, we confirmed the LC projections to spinal cord using anterograde neuronal tracing with Fluoro-ruby injected into the LC. LC reference images were obtained from the Allen Brain Atlas (https://mouse.brain-map.org/experiment/thumbnails/100048576?image_type=atlas), the spinal cord reference images were sourced from the Mouse Spinal Cord Atlas (https://mousespinal.brain-map.org/imageseries/showref.html) (Fig 5A). Following fixation, colocalization of Fluoro-ruby with TH and DBH was assessed in both LC and spinal cord tissue. Fig 5B and 5C demonstrate Fluoro-ruby-labelled neurons in the right LC and spinal dorsal horn, respectively, with TH and DBH colocalized with Fluoro-ruby. This colocalization confirmed anterograde fibers projections from LC to SDH. Neuronal activity in the SDH was further evaluated using antibodies against pCREB and c-Fos. In CIBP mice, fluorescence intensity of pCREB (*P* < 0.05 vs. sham group; Fig 5D-5E) and c-Fos-positive neurons (*P* < 0.05 vs. sham group; Fig 5F-5G) were significantly increased in SDH. Immunoblot analysis of spinal cord tissue homogenates further revealed upregulated protein levels of c-Fos (*P* < 0.05 vs. sham group) and TH (*P* < 0.05 vs. sham group) in CIBP mice (Fig 5H-5I). The results indicate that spinal cord neurons are activated in CIBP mice.

### Inhibitory neurons in spinal cord

**Fig 6:**
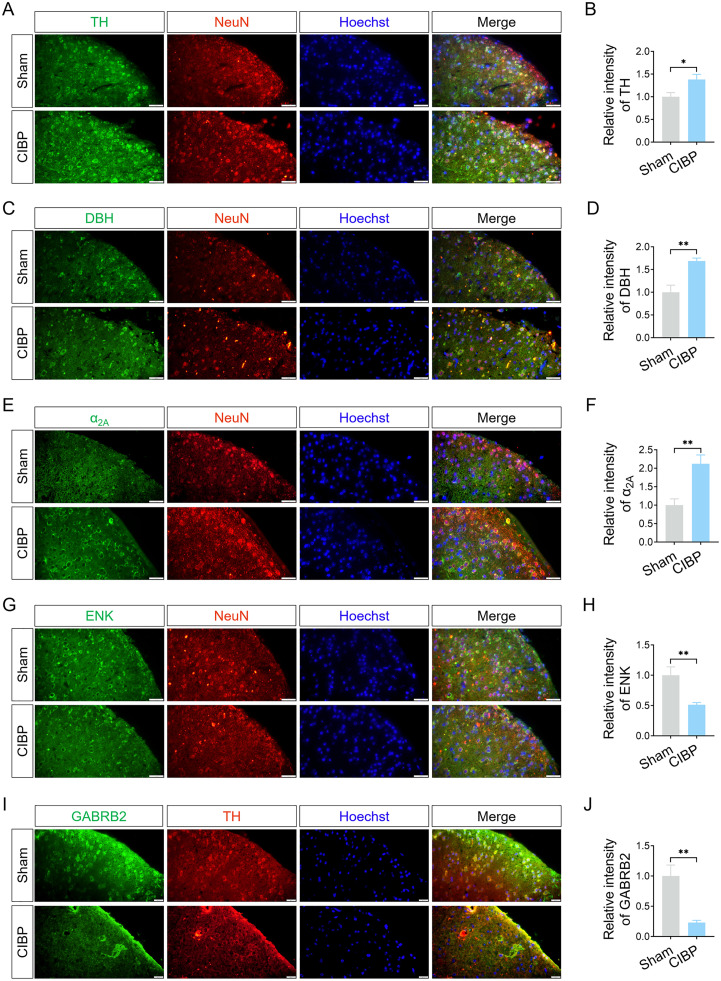
Changes in noradrenergic and inhibitory neuronal markers in the SDH. (A, C, E, G) Colocalization of TH, DBH, α2A, and ENK (green) with the neuron marker Neurn (red) in SDH. (I) Colocalization of GABRB2 (green) and TH (red) in SDH. Scale bar = 20 μm. (B, D, F, H, J) Quantitative analysis of fluorescence intensities of TH, DBH, α_2A_, ENK, and GABRB2 (n = 3). *P < 0.05, **P < 0.01 vs. sham group. Data were expressed as mean ± SD. Abbreviations: α_2A_, adrenergic receptor alpha-2A; GABRB2, GABA A receptor beta 2.

To further validate the role of LC fibers in the spinal cord, markers of noradrenergic neuron were detected in the SDH. As shown in Fig 6A, 6C, and 6E, the fluorescence intensities of TH (*P* < 0.05 vs. sham group; Fig 6B), DBH (*P *< 0.05 vs. sham group; Fig 6D) and α_2A_ receptor (*P* < 0.05 vs. sham group; Fig 6F) in the SDH of CIBP mice were significantly increased compared with sham mice. These results indicated elevated expression of noradrenergic markers in the SDH. Given that enkephalinergic and GABAergic interneurons play a role in nociceptive transmission within the spinal cord [35], the inhibitory neuron markers of enkephalin (ENK) and GABA A receptor beta 2 (GABRB2) were also examine in the SDH. The fluorescence intensities of ENK and GABRB2 were both reduced in CIBP mice relative to sham mice (Fig 6G and 6I), suggesting a decrease in inhibitory neurons following cancer cells inoculation (*P* < 0.05 vs. sham group; Fig 6H and 6J).

## Discussion

The mechanisms of CIBP include bone destruction, increased osteoclastic activity, release of inflammatory mediators, and altered sensory innervation. These mechanisms are often interlinked, reducing the efficacy of pain treatment [36]. Bone is a common site for metastasis, where tumor cells proliferate in the bone marrow, activating osteoclasts and inducing osteolysis. The resulting bone resorption releases growth factors that further promote tumor growth and exacerbate bone destruction [2]. Accordingly, the reduced trabecular bone density and elevated osteoclast numbers observed in CIBP mice (Fig 2F-2G) likely arise from tumour cells-osteoclast interactions. Nociceptive sensory neurons innervating bone respond to mechanical, thermal, and chemical stimuli [37]. In our study, spontaneous, mechanical, and thermal pain were significantly aggravated in CIBP mice (Fig 1B-1G). These findings align with progressive tumor expansion and trabecular bone degradation, both of which correlated with pain-related behaviours [38]. Furthermore, CIBP mice exhibited a correlation between pain behaviors and impaired motor coordination and balance [39]. Our results (Fig. 2C-2E) further confirmed that the increased pain sensitivity was associated with motor deficits in these mice. In summary, the pain behaviors in CIBP mice are driven by tumor-induced bone destruction.

The formation of peripheral nociceptive signals into nociception depends on pain pathways. Within these pathways, the SDH receives nociceptive signals and relays them to supraspinal centre [40]. The transmission of pain signals in the SDH is modulated by the descending pain modulatory system, which includes the PAG, LC, and RVM [41]. In this study, we investigated changes in the LC and SDH in CIBP mice. Anterograde tracing with Fluoro-ruby revealed colocalization with TH and DBH, confirming projections from LC to the SDH (Fig 5). Traditionally, the descending noradrenergic LC pathway is considered pain-inhibitory [42]. Norepinephrine release from this pathway exerts inhibitory effects via three mechanisms: Inhibition of α_2A_ adrenoceptors on primary afferent nociceptors; inhibition of spinal pain-relay neurons though α_2A_ adrenoceptors; activation of inhibitory interneurons, further dampening pain transmission [17, 43]. However, the role of the LC in pain modulation remains controversial, contrary to its classical pain-inhibitory function, the LC can also facilitation pain [18]. Noradrenergic LC terminals in the SDH contribute to allodynia and hyperalgesia in the neuropathic pain models. Selective ablation of noradrenergic neurons via intracerebroventricular anti-DBH-saporin administration reduces mechanical and cold allodynia [20], suggesting that LC-spinal cord pathway activity is critical for pain modulation. Our findings of reduced inhibitory neurons in the SDH (Fig 6G-6J) imply that the LC’s inhibitory influence on the SDH may be diminished. Further experiments – such as pharmacological manipulation of the LC-NA system and spinal inhibitory neurons- are needed to confirm this hypothesis.

Cancer-induced bone pain represents a complex pain state comprising nociceptive, inflammatory, and neuropathic components [44]. Our previous published research has demonstrated neuroinflammation in the spinal cord, characterized by glia activation and upregulation of proinflammatory cytokines [22]. Activation of the LC-spinal cord pathway can alleviate neuropathic pain by suppressing glial activation and downregulating proinflammatory cytokines expression, suggesting this pathway modulates neuroinflammation in the SDH [45]. Regarding neuropathic components, enhanced LC neuron excitability has been established across various pain models, including both inflammatory and neuropathic pain [19]. Neuropathic pain models show alterations in multiple LC proteins that correlate with pain states. In CCI rats, observed electrophysiological changes coincided with decreased pERK1/2 expression alongside increase in TH, NA transporter, and α_2A_ adrenoceptor expression in the LC [46, 47]. Similarly, DBH expression was upregulated in the LC of SNL rats [48]. Consistent with these findings, our current study detected elevated expression of noradrenergic neuron activation markers in the LC of CIBP mice.

BDNF-mediated downstream signaling pathways – including the MAPK/ERK cascade, PLCγ/DAG/PKC pathway, and PLCγ/IP3 cascade – play a key role in the persistence of in inflammatory pain, neuropathic pain, and cancer pain [49]. BDNF regulates pain in various regions of the nervous system, particularly along the pain pathway, such as the rostral anterior cingulate cortex [50], DRGs, and spinal dorsal horn [51]. In the brainstem, projections of RVM neurons releasing BDNF to inhibitory spinal cord neurons attenuate pain signalling in the spinal cord [52]. In our study, enhanced BDNF expression was observed in the LC, and BDNF- stimulated Cath.a cells exhibited increased protein levels of TH, DBH and c-Fos (Fig 4). Combined with the detection of α_2A_ adrenoceptor in the SDH (Fig 6), LC-SDH projections (Fig 5), and reduced inhibitory neuron markers (Fig 6), our results suggest that LC projections likely contributed to the pain behaviours observed in CIBP.

## Conclusions

This study investigated the expression of noradrenergic neuronal markers in the LC and SDH in a mouse model of CIBP. Our data suggest that the altered LC neuronal activity and associated regulatory proteins are linked to pain behaviours. Targeting the LC may thus provide a basis for developing novel analgesic strategies.

## Supporting information

S1 Data Raw image. (PDF)
