# Supplementary material for: Alteration of BDNF and noradrenergic markers in locus coeruleus in a mouse model of cancer-induced bone pain
Source: PLoS One. 2025 Aug 14;20(8):e0330207. doi: 10.1371/journal.pone.0330207 (PMC12352776; doi:10.1371/journal.pone.0330207)
Supplement: S1 Data — (PDF) [file pone.0330207.s001.pdf]

The original data of Western blotting in the text

Protein Ladder (26616, thermofisher)

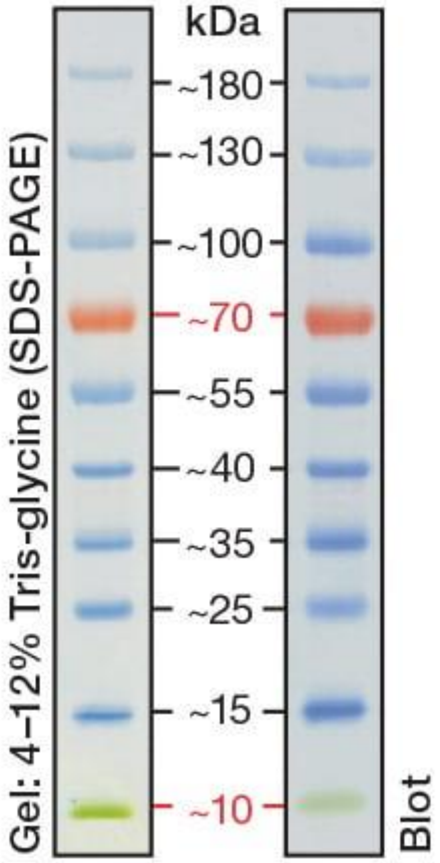

Fig 4F TH

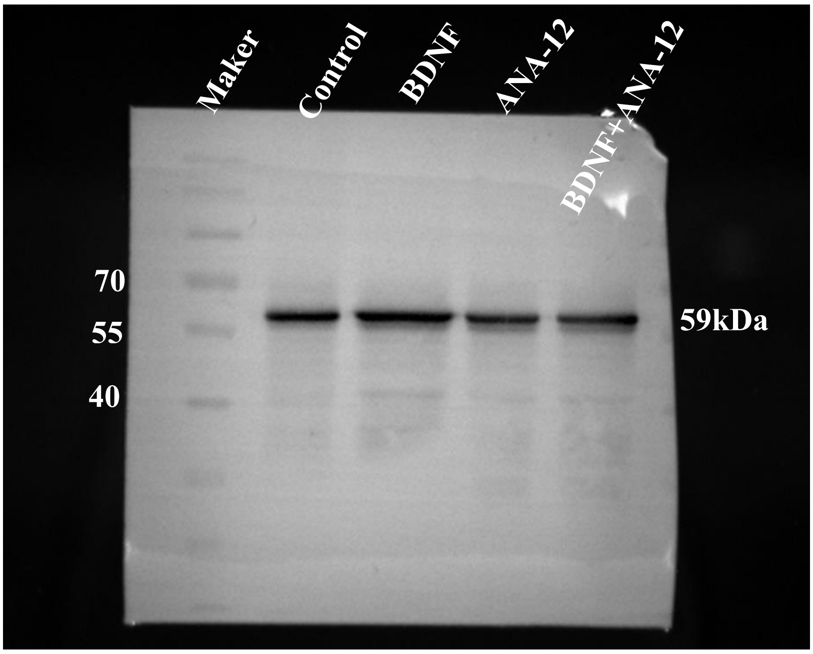

Fig 4F DBH

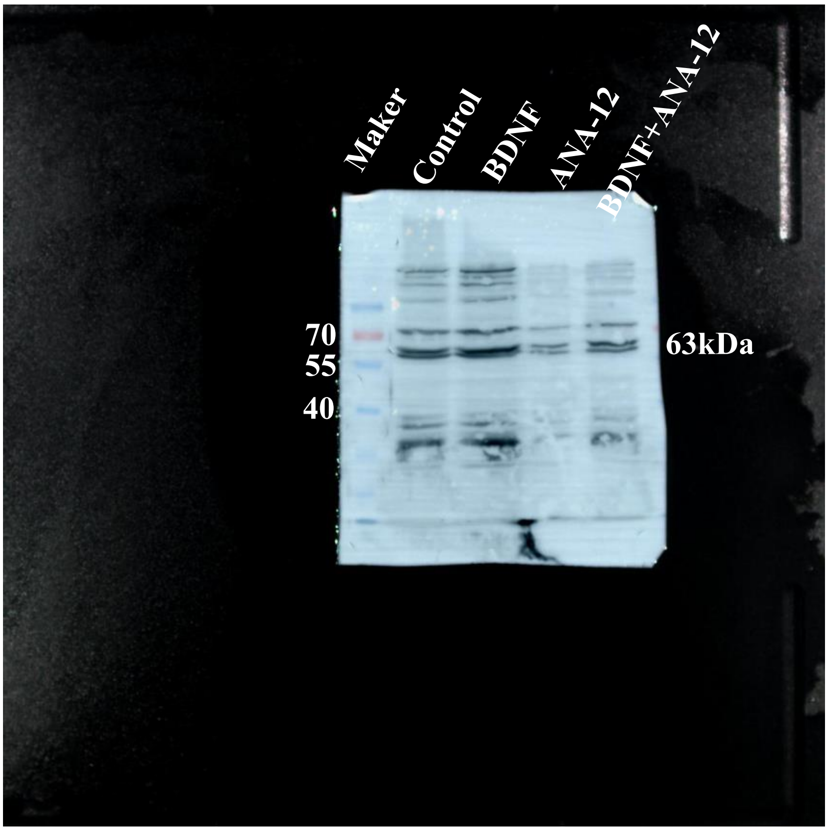

Fig 4F c-Fos

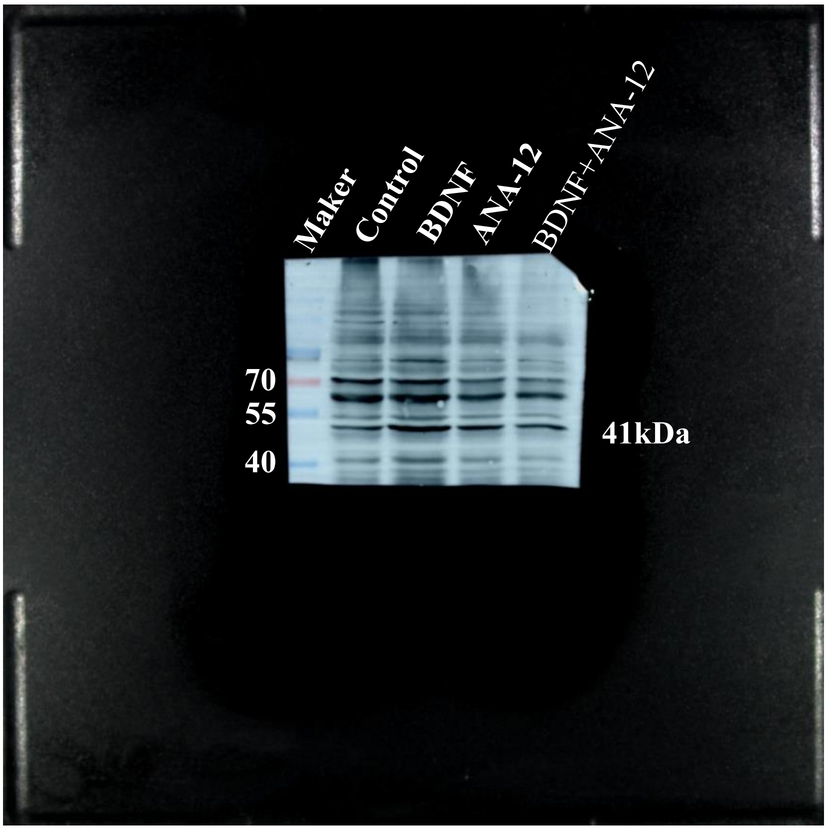

Fig 4F β-actin

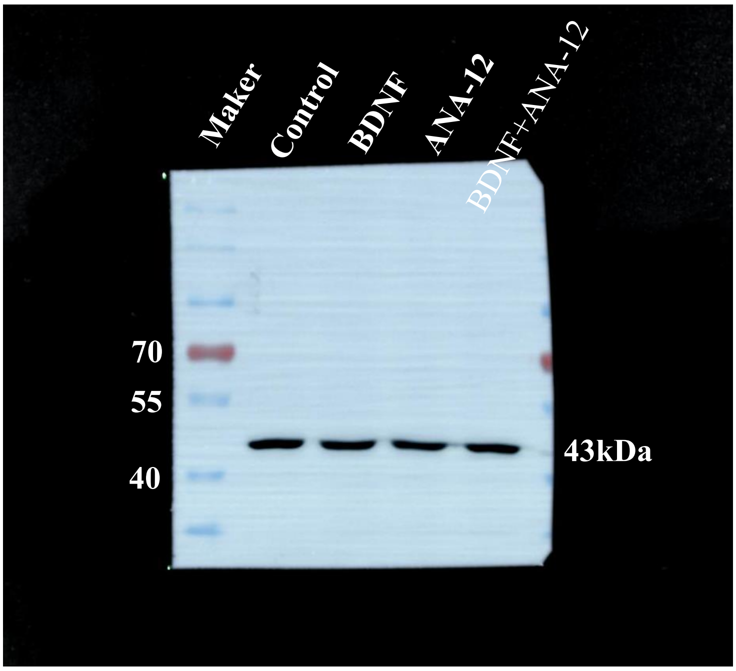

Fig 5H c-Fos

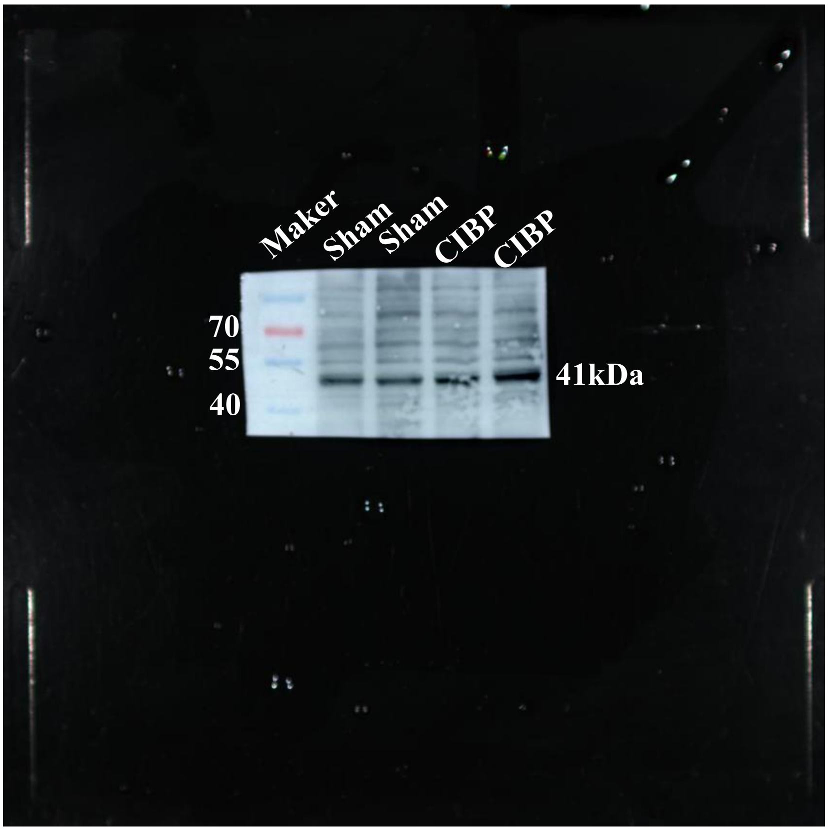

Fig 5H TH

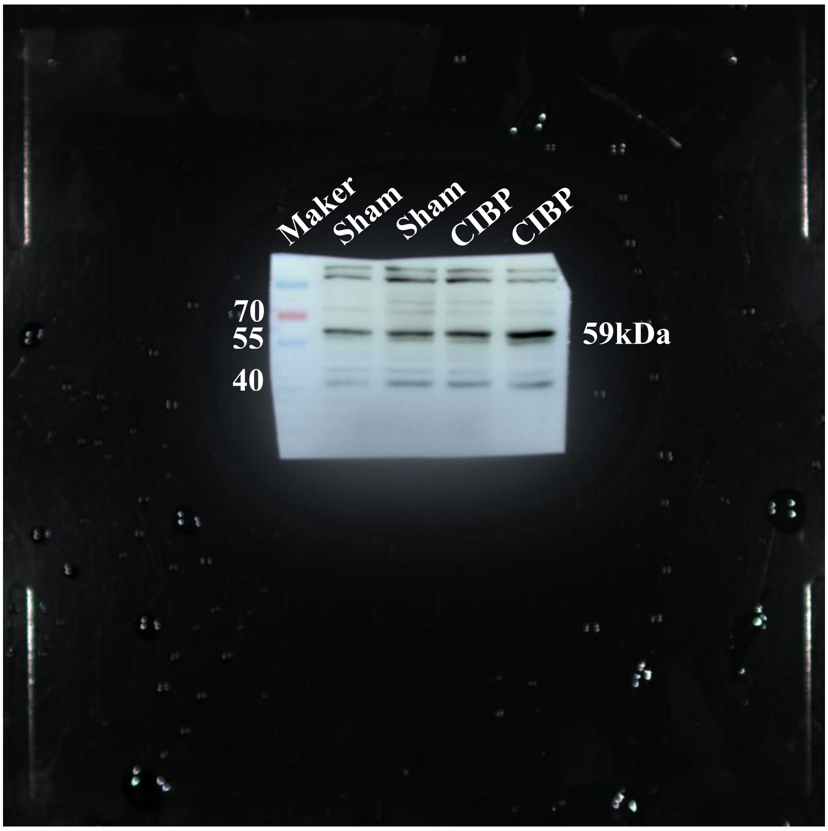

Fig 5H β-actin

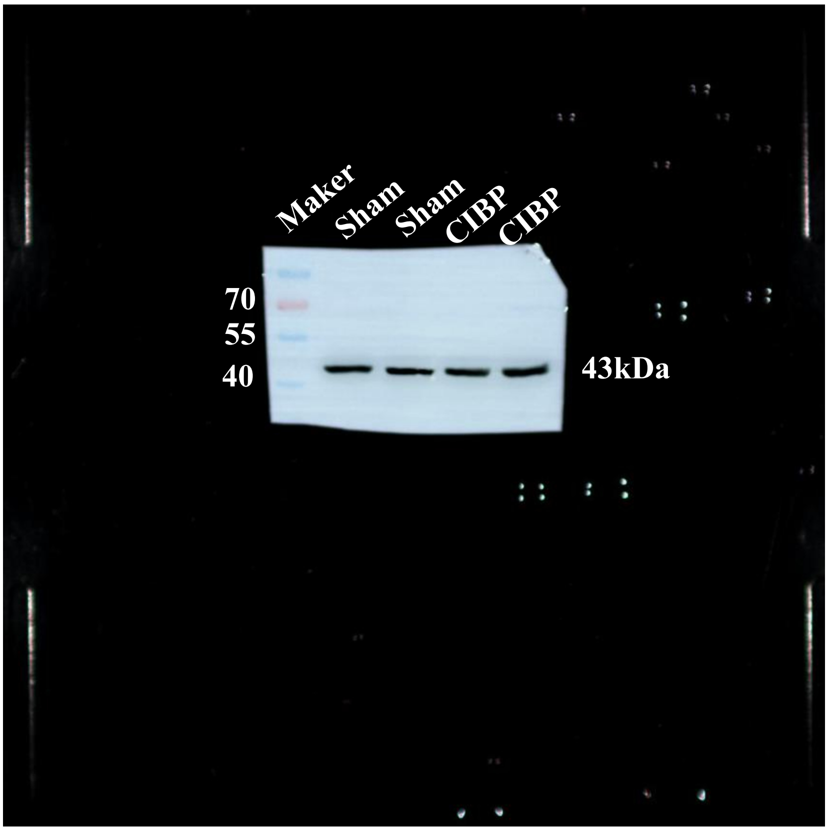

Supplementary data and statistical analysis

Fig.1C

Tests of Normality

		Kolmogorov-Smirnov[a]			Shapiro-Wilk		
	group	Statistic	df	Sig.	Statistic	df	Sig.
Flinches.0Day	Sham	.161	9	.200*	.955	9	.740
	CIBP	.223	9	.200*	.951	9	.701

*. This is a lower bound of the true significance.

a. Lilliefors Significance Correction

T-Test

Group Statistics

	group	N	Mean	Std. Deviation	Std. Error Mean
Flinches.0Day	Sham	9	2.4444	1.66667	.55556
	CIBP	9	2.2222	1.56347	.52116

Independent Samples Test

		Levene's Test for Equality of Variances		t-test for Equality of Means						
									95% Confidence Interval of the Difference	
		F	Sig.	t	df	Sig. (2-tailed)	Mean Difference	Std. Error Difference	Lower	Upper
Flinches.0Day	Equal variances assumed	.236	.634	.292	16	.774	.22222	.76174	-1.39259	1.83704
	Equal variances not assumed			.292	15.935	.774	.22222	.76174	-1.39313	1.83757

Fig.1C1

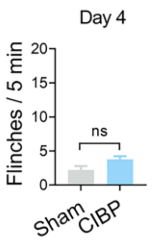

Tests of Normality

		Kolmogorov-Smirnov[a]			Shapiro-Wilk		
	group	Statistic	df	Sig.	Statistic	df	Sig.
Flinches.Day4	Sham	.125	9	.200*	.951	9	.696
	CIBP	.156	9	.200*	.938	9	.557

*. This is a lower bound of the true significance.

a. Lilliefors Significance Correction

T-Test

Group Statistics

	group	N	Mean	Std. Deviation	Std. Error Mean
Flinches.Day4	Sham	9	2.2222	1.71594	.57198
	CIBP	9	3.7778	1.39443	.46481

Independent Samples Test

		Levene's Test for Equality of Variances		t-test for Equality of Means						
									95% Confidence Interval of the Difference	
		F	Sig.	t	df	Sig. (2-tailed)	Mean Difference	Std. Error Difference	Lower	Upper
Flinches.Day4	Equal variances assumed	.326	.576	-2.111	16	.051	-1.55556	.73703	-3.11798	.00687
	Equal variances not assumed			-2.111	15.357	.052	-1.55556	.73703	-3.12331	.01220

Fig.1C2

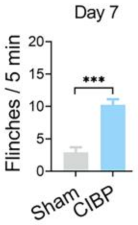

Tests of Normality

	group	Kolmogorov-Smirnov[a] Statistic	df	Sig.	Shapiro-Wilk Statistic	df	Sig.
Flinches.Day7	Sham	.199	9	.200*	.890	9	.200
	CIBP	.133	9	.200*	.971	9	.903

*. This is a lower bound of the true significance.

a. Lilliefors Significance Correction

T-Test

Group Statistics

	group	N	Mean	Std. Deviation	Std. Error Mean
Flinches.Day7	Sham	9	2.8889	2.42097	.80699
	CIBP	9	10.2222	2.63523	.87841

Independent Samples Test

		Levene's Test for Equality of Variances		t-test for Equality of Means					95% Confidence Interval of the Difference	
		F	Sig.	t	df	Sig. (2-tailed)	Mean Difference	Std. Error Difference	Lower	Upper
Flinches.Day7	Equal variances assumed	.015	.903	-6.148	16	.000	-7.33333	1.19283	-9.86202	-4.80465
	Equal variances not assumed			-6.148	15.886	.000	-7.33333	1.19283	-9.86349	-4.80318

Fig.1C3

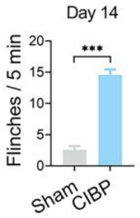

Tests of Normality

	group	Kolmogorov-Smirnov[a] Statistic	df	Sig.	Shapiro-Wilk Statistic	df	Sig.
Flinches.Day14	Sham	.181	9	.200*	.900	9	.252
	CIBP	.166	9	.200*	.908	9	.301

*. This is a lower bound of the true significance.

a. Lilliefors Significance Correction

T-Test

Group Statistics

	group	N	Mean	Std. Deviation	Std. Error Mean
Flinches.Day14	Sham	9	2.5556	1.81046	.60349
	CIBP	9	14.5556	2.65100	.88367

Independent Samples Test

		Levene's Test for Equality of Variances		t-test for Equality of Means					95% Confidence Interval of the Difference	
		F	Sig.	t	df	Sig. (2-tailed)	Mean Difference	Std. Error Difference	Lower	Upper
Flinches.Day14	Equal variances assumed	3.027	.101	-11.214	16	.000	-12.00000	1.07008	-14.26846	-9.73154
	Equal variances not assumed			-11.214	14.129	.000	-12.00000	1.07008	-14.29312	-9.70688

Fig.1E

Tests of Normality

	group	Kolmogorov-Smirnov[a] Statistic	df	Sig.	Shapiro-Wilk Statistic	df	Sig.
PWT.Day0	Sham	.229	9	.190	.896	9	.232
	CIBP	.192	9	.200*	.896	9	.232

*. This is a lower bound of the true significance.

a. Lilliefors Significance Correction

T-Test

Group Statistics

	group	N	Mean	Std. Deviation	Std. Error Mean
PWT.Day0	Sham	9	1.238380689	.4978587316	.1659529105
	CIBP	9	1.350451104	.3752640760	.1250880253

Independent Samples Test

		Levene's Test for Equality of Variances		t-test for Equality of Means					95% Confidence Interval of the Difference	
		F	Sig.	t	df	Sig. (2-tailed)	Mean Difference	Std. Error Difference	Lower	Upper
PWT.Day0	Equal variances assumed	.002	.963	-.539	16	.597	-.1120704149	.2078157420	-.5526201075	.3284792777
	Equal variances not assumed			-.539	14.872	.598	-.1120704149	.2078157420	-.5553511771	.3312103473

Fig.1E1

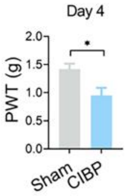

Tests of Normality

	group	Kolmogorov-Smirnov[a] Statistic	df	Sig.	Shapiro-Wilk Statistic	df	Sig.
PWT.Day4	Sham	.196	9	.200*	.951	9	.700
	CIBP	.158	9	.200*	.941	9	.598

*. This is a lower bound of the true significance.

a. Lilliefors Significance Correction

T-Test

Group Statistics

	group	N	Mean	Std. Deviation	Std. Error Mean
PWT.Day4	Sham	9	1.418747470	.2929895684	.0976631895
	CIBP	9	.950409434	.4043133572	.1347711191

Independent Samples Test

		Levene's Test for Equality of Variances		t-test for Equality of Means					95% Confidence Interval of the Difference	
		F	Sig.	t	df	Sig. (2-tailed)	Mean Difference	Std. Error Difference	Lower	Upper
PWT.Day4	Equal variances assumed	.305	.588	2.814	16	.012	.4683380368	.1664372348	.1155068608	.8211692127
	Equal variances not assumed			2.814	14.586	.013	.4683380368	.1664372348	.1127063433	.8239697303

Fig.1E2

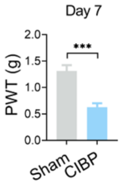

Tests of Normality

	group	Kolmogorov-Smirnov[a] Statistic	df	Sig.	Shapiro-Wilk Statistic	df	Sig.
PWT.Day7	Sham	.217	9	.200*	.905	9	.282
	CIBP	.188	9	.200*	.929	9	.469

*. This is a lower bound of the true significance.

a. Lilliefors Significance Correction

T-Test

Group Statistics

	group	N	Mean	Std. Deviation	Std. Error Mean
PWT.Day7	Sham	9	1.313543305	.3250226325	.1083408775
	CIBP	9	.627975861	.2190642914	.0730214305

Independent Samples Test

		Levene's Test for Equality of Variances F	Sig.	t-test for Equality of Means t	df	Sig. (2-tailed)	Mean Difference	Std. Error Difference	95% Confidence Interval of the Difference Lower	Upper
PWT.Day7	Equal variances assumed	3.759	.070	5.247	16	.000	.6855674436	.1306517319	.4085981448	.9625367424
	Equal variances not assumed			5.247	14.025	.000	.6855674436	.1306517319	.4053942251	.9657406620

Fig.1E3

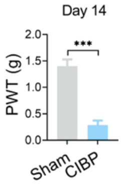

Tests of Normality

	group	Kolmogorov-Smirnov[a] Statistic	df	Sig.	Shapiro-Wilk Statistic	df	Sig.
PWT.Day14	Sham	.213	9	.200*	.949	9	.674
	CIBP	.164	9	.200*	.928	9	.462

*. This is a lower bound of the true significance.

a. Lilliefors Significance Correction

T-Test

Group Statistics

	group	N	Mean	Std. Deviation	Std. Error Mean
PWT.Day14	Sham	9	1.397044889	.3908611724	.1302870575
	CIBP	9	.286308572	.2531376989	.0843792330

Independent Samples Test

		Levene's Test for Equality of Variances F	Sig.	t-test for Equality of Means t	df	Sig. (2-tailed)	Mean Difference	Std. Error Difference	95% Confidence Interval of the Difference Lower	Upper
PWT.Day14	Equal variances assumed	2.454	.137	7.156	16	.000	1.110736318	.1552242645	.7816755767	1.439797059
	Equal variances not assumed			7.156	13.707	.000	1.110736318	.1552242645	.7771444826	1.444328153

Fig.1G

Tests of Normality

	group	Kolmogorov-Smirnov[a] Statistic	df	Sig.	Shapiro-Wilk Statistic	df	Sig.
PWL.Day0	Sham	.209	9	.200*	.946	9	.645
	CIBP	.132	9	.200*	.943	9	.615

*. This is a lower bound of the true significance.

a. Lilliefors Significance Correction

T-Test

Group Statistics

	group	N	Mean	Std. Deviation	Std. Error Mean
PWL.Day0	Sham	9	13.74222	2.121826	.707275
	CIBP	9	13.54000	1.807395	.602465

Independent Samples Test

		Levene's Test for Equality of Variances		t-test for Equality of Means						
									95% Confidence Interval of the Difference	
		F	Sig.	t	df	Sig. (2-tailed)	Mean Difference	Std. Error Difference	Lower	Upper
PWL.Day0	Equal variances assumed	.439	.517	.218	16	.830	.202222	.929087	-1.767354	2.171798
	Equal variances not assumed			.218	15.605	.831	.202222	.929087	-1.771410	2.175854

Fig.1G1

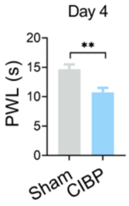

Tests of Normality

	group	Kolmogorov-Smirnov[a] Statistic	df	Sig.	Shapiro-Wilk Statistic	df	Sig.
PWL.Day4	Sham	.132	9	.200*	.968	9	.879
	CIBP	.123	9	.200*	.983	9	.979

*. This is a lower bound of the true significance.

a. Lilliefors Significance Correction

T-Test

Group Statistics

	group	N	Mean	Std. Deviation	Std. Error Mean
PWL.Day4	Sham	9	14.65111	2.539879	.846626
	CIBP	9	10.73333	2.304159	.768053

Independent Samples Test

		Levene's Test for Equality of Variances		t-test for Equality of Means						
									95% Confidence Interval of the Difference	
		F	Sig.	t	df	Sig. (2-tailed)	Mean Difference	Std. Error Difference	Lower	Upper
PWL.Day4	Equal variances assumed	.158	.697	3.427	16	.003	3.917778	1.143102	1.494510	6.341045
	Equal variances not assumed			3.427	15.851	.003	3.917778	1.143102	1.492652	6.342903

Fig.1G2

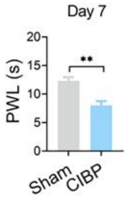

Tests of Normality

	group	Kolmogorov-Smirnov[a] Statistic	df	Sig.	Shapiro-Wilk Statistic	df	Sig.
PWL.Day7	Sham	.133	9	.200*	.963	9	.826
	CIBP	.117	9	.200*	.960	9	.797

*. This is a lower bound of the true significance.

a. Lilliefors Significance Correction

T-Test

Group Statistics

	group	N	Mean	Std. Deviation	Std. Error Mean
PWL.Day7	Sham	9	12.30778	1.965437	.655146
	CIBP	9	7.99889	2.247279	.749093

Independent Samples Test

		Levene's Test for Equality of Variances		t-test for Equality of Means						
									95% Confidence Interval of the Difference	
		F	Sig.	t	df	Sig. (2-tailed)	Mean Difference	Std. Error Difference	Lower	Upper
PWL.Day7	Equal variances assumed	.117	.737	4.330	16	.001	4.308889	.995166	2.199230	6.418547
	Equal variances not assumed			4.330	15.721	.001	4.308889	.995166	2.196184	6.421594

Fig.1G3

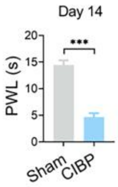

Tests of Normality

	group	Kolmogorov-Smirnov[a] Statistic	df	Sig.	Shapiro-Wilk Statistic	df	Sig.
PWL.Day14	Sham	.171	9	.200*	.946	9	.649
	CIBP	.189	9	.200*	.929	9	.468

*. This is a lower bound of the true significance.

a. Lilliefors Significance Correction

T-Test

Group Statistics

	group	N	Mean	Std. Deviation	Std. Error Mean
PWL.Day14	Sham	9	14.41889	2.758525	.919508
	CIBP	9	4.66556	2.198858	.732953

Independent Samples Test

		Levene's Test for Equality of Variances		t-test for Equality of Means						
									95% Confidence Interval of the Difference	
		F	Sig.	t	df	Sig. (2-tailed)	Mean Difference	Std. Error Difference	Lower	Upper
PWL.Day14	Equal variances assumed	.832	.375	8.294	16	.000	9.753333	1.175889	7.260560	12.246107
	Equal variances not assumed			8.294	15.242	.000	9.753333	1.175889	7.250451	12.256215

Fig. 2B

Tests of Normality

	group	Kolmogorov-Smirnov[a] Statistic	df	Sig.	Shapiro-Wilk Statistic	df	Sig.
Latency.on.rotarod.Day0	Sham	.227	9	.200*	.872	9	.128
	CIBP	.189	9	.200*	.882	9	.165

*. This is a lower bound of the true significance.

a. Lilliefors Significance Correction

T-Test

Group Statistics

	group	N	Mean	Std. Deviation	Std. Error Mean
Latency.on.rotarod.Day0	Sham	9	545.84667	58.223467	19.407822
	CIBP	9	537.61778	59.976472	19.992157

Independent Samples Test

		Levene's Test for Equality of Variances		t-test for Equality of Means						
									95% Confidence Interval of the Difference	
		F	Sig.	t	df	Sig. (2-tailed)	Mean Difference	Std. Error Difference	Lower	Upper
Latency.on.rotarod.Day0	Equal variances assumed	.007	.936	.295	16	.772	8.228889	27.863057	-50.838152	67.295930
	Equal variances not assumed			.295	15.986	.772	8.228889	27.863057	-50.842374	67.300152

Fig. 2B1

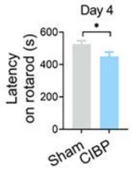

Tests of Normality

	group	Kolmogorov-Smirnov[a] Statistic	df	Sig.	Shapiro-Wilk Statistic	df	Sig.
Latency.on.rotarod.Day4	Sham	.190	9	.200*	.922	9	.411
	CIBP	.138	9	.200*	.939	9	.567

*. This is a lower bound of the true significance.

a. Lilliefors Significance Correction

T-Test

Group Statistics

	group	N	Mean	Std. Deviation	Std. Error Mean
Latency.on.rotarod.Day4	Sham	9	524.64889	66.647828	22.215943
	CIBP	9	449.96556	79.707724	26.569241

Independent Samples Test

		Levene's Test for Equality of Variances		t-test for Equality of Means						
									95% Confidence Interval of the Difference	
		F	Sig.	t	df	Sig. (2-tailed)	Mean Difference	Std. Error Difference	Lower	Upper
Latency.on.rotarod.Day4	Equal variances assumed	.258	.619	2.156	16	.047	74.683333	34.633404	1.263796	148.102870
	Equal variances not assumed			2.156	15.514	.047	74.683333	34.633404	1.076288	148.290379

Fig. 2B2

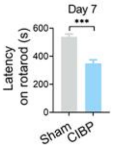

Tests of Normality

	group	Kolmogorov-Smirnov[a] Statistic	df	Sig.	Shapiro-Wilk Statistic	df	Sig.
Latency.on.rotarod.Day7	Sham	.204	9	.200*	.900	9	.249
	CIBP	.153	9	.200*	.943	9	.612

*. This is a lower bound of the true significance.

a. Lilliefors Significance Correction

T-Test

Group Statistics

	group	N	Mean	Std. Deviation	Std. Error Mean
Latency.on.rotarod.Day7	Sham	9	538.28556	60.589018	20.196339
	CIBP	9	348.57444	77.334282	25.778094

Independent Samples Test

		Levene's Test for Equality of Variances		t-test for Equality of Means					95% Confidence Interval of the Difference	
		F	Sig.	t	df	Sig. (2-tailed)	Mean Difference	Std. Error Difference	Lower	Upper
Latency.on.rotarod.Day7	Equal variances assumed	.081	.780	5.793	16	.000	189.711111	32.747553	120.289399	259.132823
	Equal variances not assumed			5.793	15.133	.000	189.711111	32.747553	119.964922	259.457301

Fig. 2B3

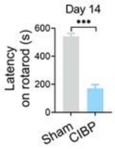

Tests of Normality

	group	Kolmogorov-Smirnov[a] Statistic	df	Sig.	Shapiro-Wilk Statistic	df	Sig.
Latency.on.rotarod.Day14	Sham	.217	9	.200*	.862	9	.101
	CIBP	.179	9	.200*	.915	9	.350

*. This is a lower bound of the true significance.

a. Lilliefors Significance Correction

T-Test

Group Statistics

	group	N	Mean	Std. Deviation	Std. Error Mean
Latency.on.rotarod.Day14	Sham	9	540.76778	63.949448	21.316483
	CIBP	9	171.41222	77.481938	25.827313

Independent Samples Test

		Levene's Test for Equality of Variances		t-test for Equality of Means					95% Confidence Interval of the Difference	
		F	Sig.	t	df	Sig. (2-tailed)	Mean Difference	Std. Error Difference	Lower	Upper
Latency.on.rotarod.Day14	Equal variances assumed	.051	.824	11.030	16	.000	369.355556	33.487946	298.364282	440.346829
	Equal variances not assumed			11.030	15.445	.000	369.355556	33.487946	298.156244	440.554867

Fig. 2C

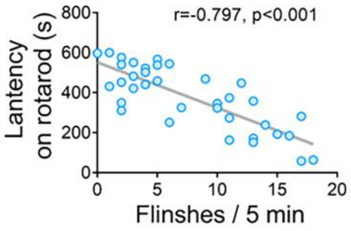

Correlations

		Lantency.on. rotarod	Flinshes
Lantency.on.rotarod	Pearson Correlation	1	-.797**
	Sig. (2-tailed)		.000
	N	36	36
Flinshes	Pearson Correlation	-.797**	1
	Sig. (2-tailed)	.000	
	N	36	36

**. Correlation is significant at the 0.01 level (2-tailed).

Fig. 2D

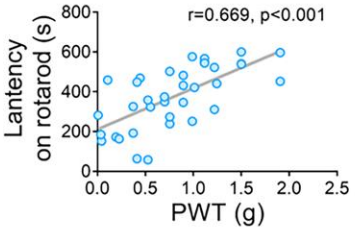

Correlations

		Lantency.on. rotarod	PWT
Lantency.on.rotarod	Pearson Correlation	1	.669**
	Sig. (2-tailed)		.000
	N	36	36
PWT	Pearson Correlation	.669**	1
	Sig. (2-tailed)	.000	
	N	36	36

**. Correlation is significant at the 0.01 level (2-tailed).

Fig. 2E

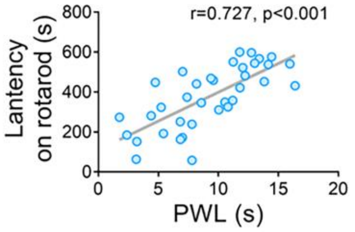

Correlations

		Lantency.on. rotarod	PWL
Lantency.on.rotarod	Pearson Correlation	1	.727**
	Sig. (2-tailed)		.000
	N	36	36
PWL	Pearson Correlation	.727**	1
	Sig. (2-tailed)	.000	
	N	36	36

**. Correlation is significant at the 0.01 level (2-tailed).

Fig.2F

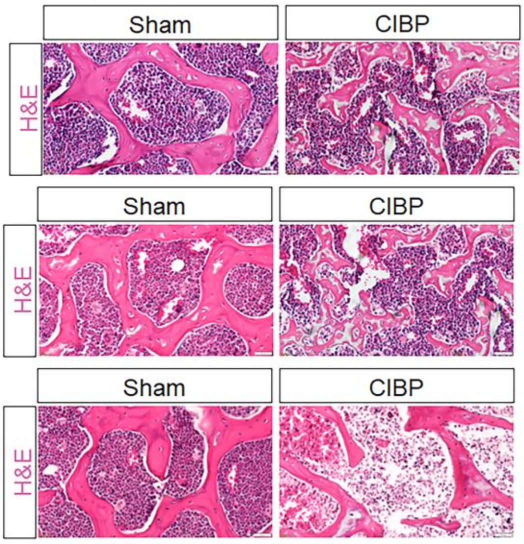

Fig.2G

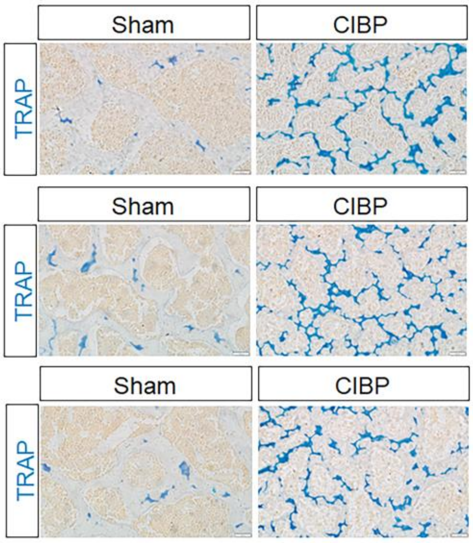

Fig.2H

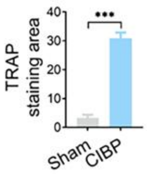

Tests of Normality

	group	Kolmogorov-Smirnov[a] Statistic	Kolmogorov-Smirnov[a] df	Kolmogorov-Smirnov[a] Sig.	Shapiro-Wilk Statistic	Shapiro-Wilk df	Shapiro-Wilk Sig.
Trap	Sham	.245	3	.	.971	3	.672
	CIBP	.188	3	.	.998	3	.912

a. Lilliefors Significance Correction

T-Test

Group Statistics

	group	N	Mean	Std. Deviation	Std. Error Mean
Trap	Sham	3	3.28433	1.095637	.632566
	CIBP	3	30.96567	1.934045	1.116621

Independent Samples Test

		Levene's Test for Equality of Variances		t-test for Equality of Means					95% Confidence Interval of the Difference	
		F	Sig.	t	df	Sig. (2-tailed)	Mean Difference	Std. Error Difference	Lower	Upper
Trap	Equal variances assumed	.612	.478	-21.570	4	.000	-27.681333	1.283348	-31.244480	-24.118187
	Equal variances not assumed			-21.570	3.164	.000	-27.681333	1.283348	-31.648448	-23.714219

Fig.3B

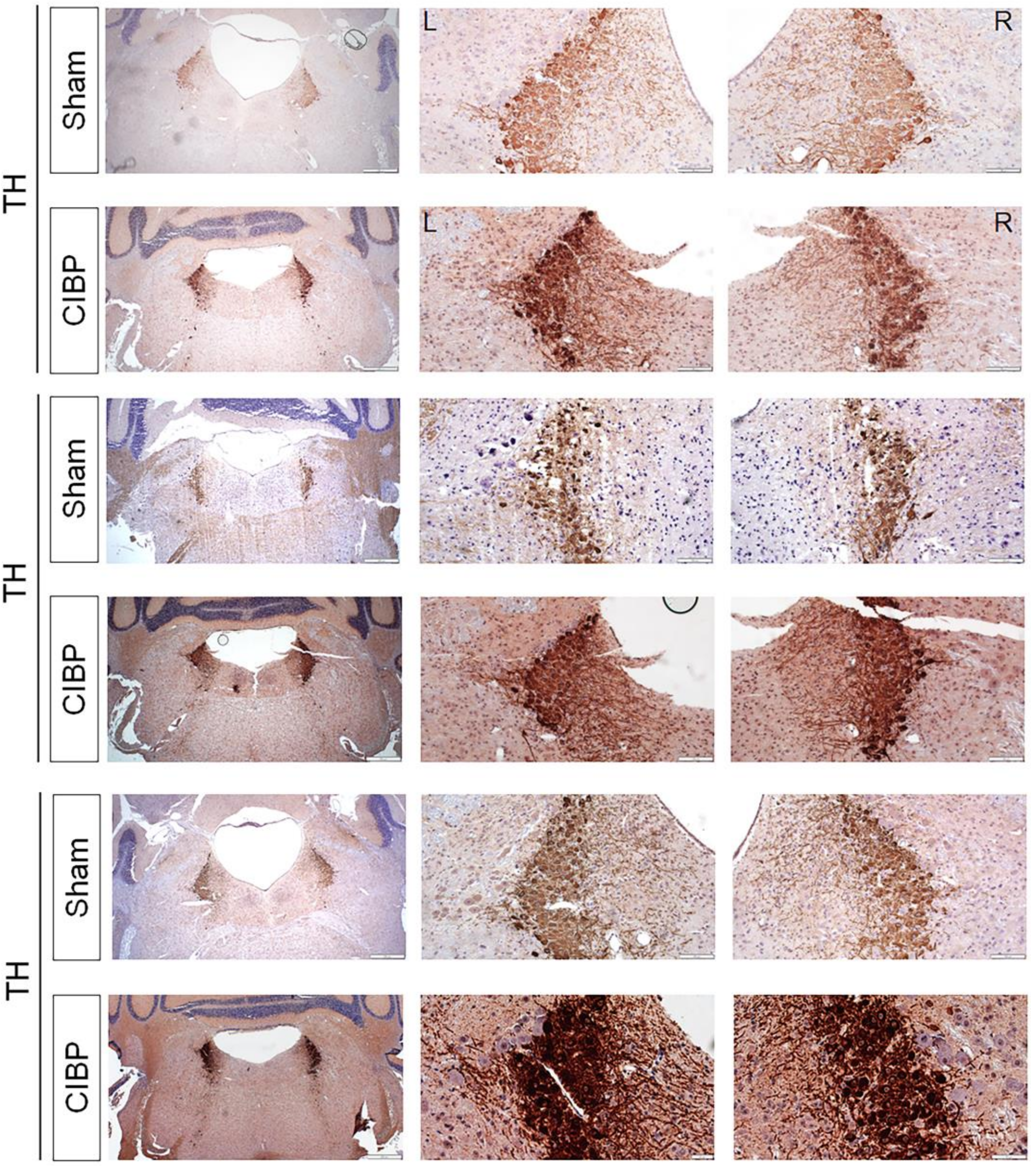

Fig.3C

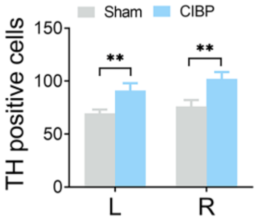

Tests of Normality

	group	Kolmogorov-Smirnov[a] Statistic	df	Sig.	Shapiro-Wilk Statistic	df	Sig.
LC.TH.L	Sham	.204	3	.	.993	3	.843
	CIBP	.175	3	.	1.000	3	1.000

a. Lilliefors Significance Correction

T-Test

Group Statistics

	group	N	Mean	Std. Deviation	Std. Error Mean
LC.TH.L	Sham	3	69.66667	3.511885	2.027588
	CIBP	3	91.00000	7.000000	4.041452

Independent Samples Test

		Levene's Test for Equality of Variances		t-test for Equality of Means						
									95% Confidence Interval of the Difference	
		F	Sig.	t	df	Sig. (2-tailed)	Mean Difference	Std. Error Difference	Lower	Upper
LC.TH.L	Equal variances assumed	.752	.435	-4.718	4	.009	-21.333333	4.521553	-33.887178	-8.779489
	Equal variances not assumed			-4.718	2.947	.019	-21.333333	4.521553	-35.870967	-6.795699

Tests of Normality

	group	Kolmogorov-Smirnov[a] Statistic	df	Sig.	Shapiro-Wilk Statistic	df	Sig.
LC.TH.R	Sham	.292	3	.	.923	3	.463
	CIBP	.227	3	.	.983	3	.747

a. Lilliefors Significance Correction

T-Test

Group Statistics

	group	N	Mean	Std. Deviation	Std. Error Mean
LC.TH.R	Sham	3	76.0000	6.24500	3.60555
	CIBP	3	102.0000	6.55744	3.78594

Independent Samples Test

		Levene's Test for Equality of Variances		t-test for Equality of Means						
									95% Confidence Interval of the Difference	
		F	Sig.	t	df	Sig. (2-tailed)	Mean Difference	Std. Error Difference	Lower	Upper
LC.TH.R	Equal variances assumed	.000	1.000	-4.973	4	.008	-26.00000	5.22813	-40.51561	-11.48439
	Equal variances not assumed			-4.973	3.991	.008	-26.00000	5.22813	-40.52925	-11.47075

Fig.3D

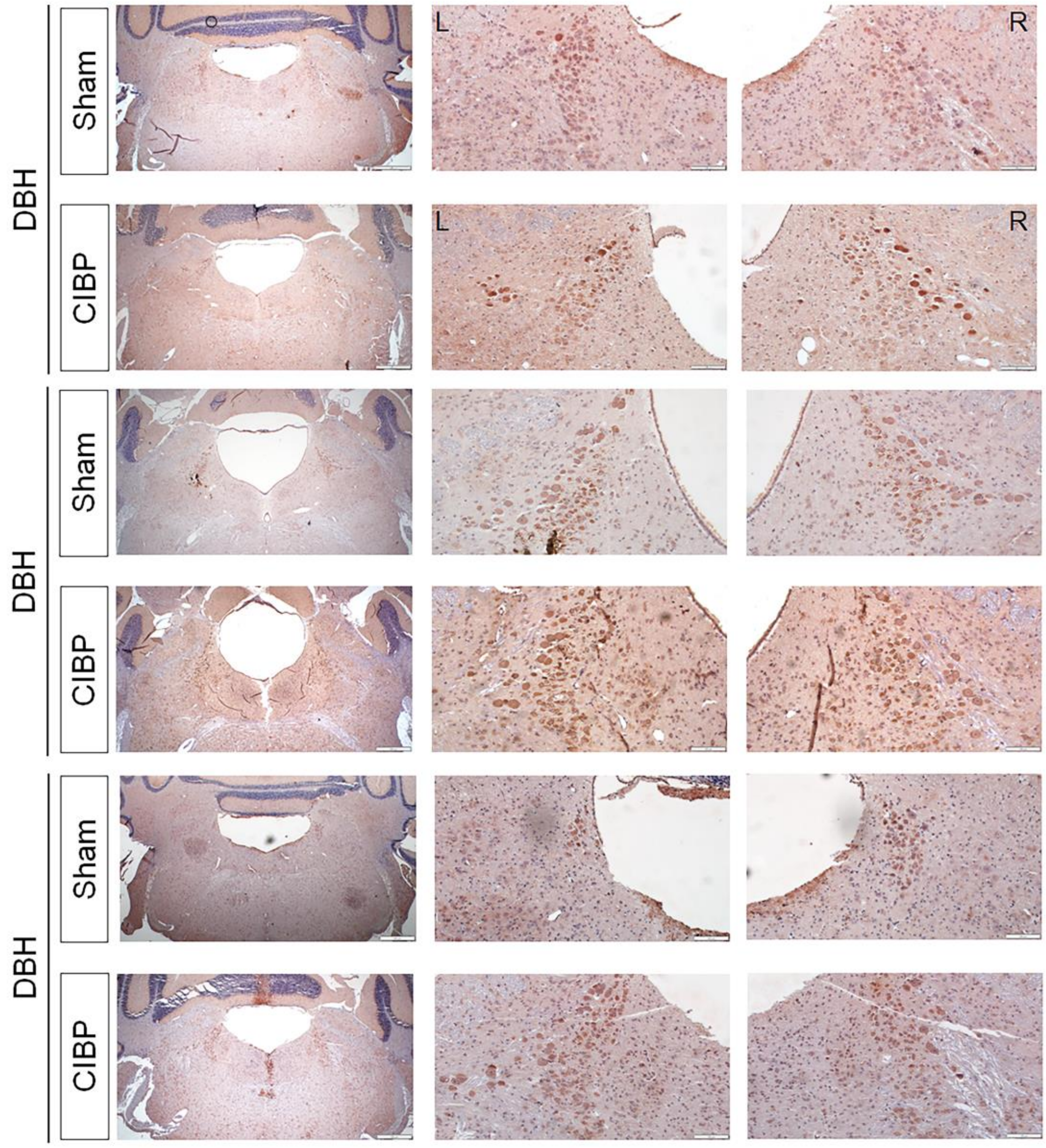

Fig.3E

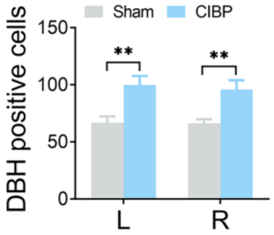

Tests of Normality

	group	Kolmogorov-Smirnov[a] Statistic	df	Sig.	Shapiro-Wilk Statistic	df	Sig.
LC.DBH.L	Sham	.191	3	.	.997	3	.900
	CIBP	.200	3	.	.995	3	.862

a. Lilliefors Significance Correction

T-Test

Group Statistics

	group	N	Mean	Std. Deviation	Std. Error Mean
LC.DBH.L	Sham	3	66.6667	5.50757	3.17980
	CIBP	3	99.6667	8.02081	4.63081

Independent Samples Test

		Levene's Test for Equality of Variances		t-test for Equality of Means						
									95% Confidence Interval of the Difference	
		F	Sig.	t	df	Sig. (2-tailed)	Mean Difference	Std. Error Difference	Lower	Upper
LC.DBH.L	Equal variances assumed	.352	.585	-5.875	4	.004	-33.00000	5.61743	-48.59649	-17.40351
	Equal variances not assumed			-5.875	3.543	.006	-33.00000	5.61743	-49.42288	-16.57712

Tests of Normality

	group	Kolmogorov-Smirnov[a] Statistic	df	Sig.	Shapiro-Wilk Statistic	df	Sig.
LC.DBH.R	Sham	.204	3	.	.993	3	.843
	CIBP	.292	3	.	.923	3	.463

a. Lilliefors Significance Correction

T-Test

Group Statistics

	group	N	Mean	Std. Deviation	Std. Error Mean
LC.DBH.R	Sham	3	66.3333	3.51188	2.02759
	CIBP	3	95.6667	8.32666	4.80740

Independent Samples Test

		Levene's Test for Equality of Variances		t-test for Equality of Means						
									95% Confidence Interval of the Difference	
		F	Sig.	t	df	Sig. (2-tailed)	Mean Difference	Std. Error Difference	Lower	Upper
LC.DBH.R	Equal variances assumed	2.927	.162	-5.622	4	.005	-29.33333	5.21749	-43.81941	-14.84725
	Equal variances not assumed			-5.622	2.690	.015	-29.33333	5.21749	-47.07575	-11.59092

Fig.3F

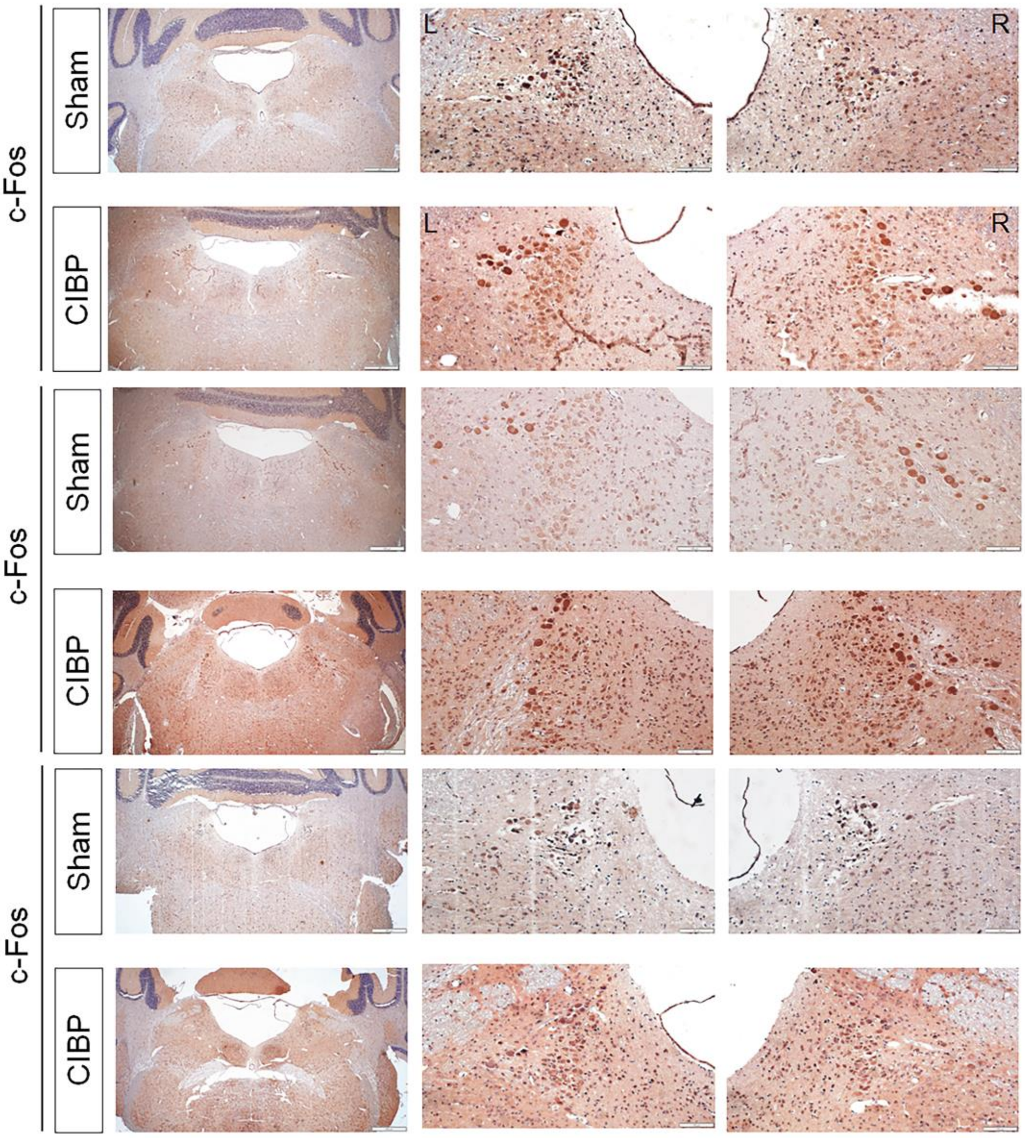

Fig.3G

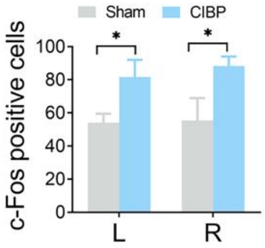

Tests of Normality

	group	Kolmogorov-Smirnov[a] Statistic	df	Sig.	Shapiro-Wilk Statistic	df	Sig.
LC.cFos.L	Sham	.238	3	.	.976	3	.702
	CIBP	.292	3	.	.923	3	.463

a. Lilliefors Significance Correction

T-Test

Group Statistics

	group	N	Mean	Std. Deviation	Std. Error Mean
LC.cFos.L	Sham	3	54.0000	5.56776	3.21455
	CIBP	3	81.6667	10.40833	6.00925

Independent Samples Test

		Levene's Test for Equality of Variances		t-test for Equality of Means					95% Confidence Interval of the Difference	
		F	Sig.	t	df	Sig. (2-tailed)	Mean Difference	Std. Error Difference	Lower	Upper
LC.cFos.L	Equal variances assumed	1.741	.257	-4.060	4	.015	-27.66667	6.81502	-46.58818	-8.74515
	Equal variances not assumed			-4.060	3.058	.026	-27.66667	6.81502	-49.12429	-6.20905

Tests of Normality

	group	Kolmogorov-Smirnov[a] Statistic	df	Sig.	Shapiro-Wilk Statistic	df	Sig.
LC.cFos.R	Sham	.301	3	.	.912	3	.424
	CIBP	.282	3	.	.936	3	.510

a. Lilliefors Significance Correction

T-Test

Group Statistics

	group	N	Mean	Std. Deviation	Std. Error Mean
LC.cFos.R	Sham	3	55.3333	13.61372	7.85988
	CIBP	3	88.3333	5.68624	3.28295

Independent Samples Test

		Levene's Test for Equality of Variances		t-test for Equality of Means					95% Confidence Interval of the Difference	
		F	Sig.	t	df	Sig. (2-tailed)	Mean Difference	Std. Error Difference	Lower	Upper
LC.cFos.R	Equal variances assumed	3.159	.150	-3.874	4	.018	-33.00000	8.51795	-56.64963	-9.35037
	Equal variances not assumed			-3.874	2.677	.037	-33.00000	8.51795	-62.05396	-3.94604

Fig.4A

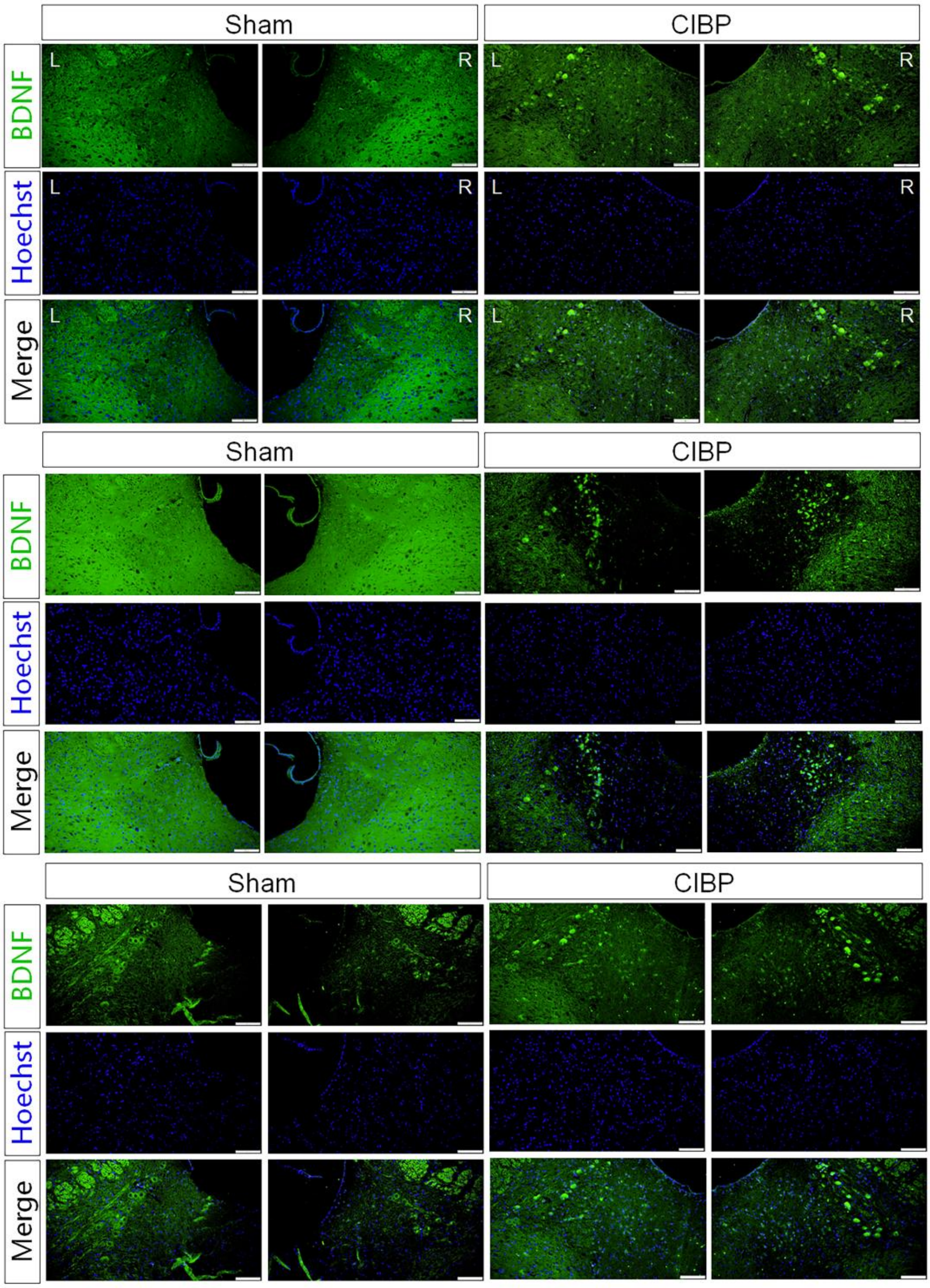
Sham
CIBP
BDNF
Hoechst
Merge
L
R
Sham
CIBP
BDNF
Hoechst
Merge
Sham
CIBP
BDNF
Hoechst
Merge

Fig.4B

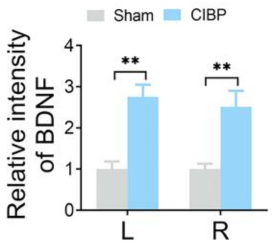

Tests of Normality

	group	Kolmogorov-Smirnov[a] Statistic	df	Sig.	Shapiro-Wilk Statistic	df	Sig.
LC.BDNF.L	Sham	.210	3	.	.991	3	.821
	CIBP	.273	3	.	.946	3	.551

a. Lilliefors Significance Correction

T-Test

Group Statistics

	group	N	Mean	Std. Deviation	Std. Error Mean
LC.BDNF.L	Sham	3	1.000000000	.1893884204	.1093434555
	CIBP	3	2.753905966	.2937641254	.1696047969

Independent Samples Test

		Levene's Test for Equality of Variances		t-test for Equality of Means						
									95% Confidence Interval of the Difference	
		F	Sig.	t	df	Sig. (2-tailed)	Mean Difference	Std. Error Difference	Lower	Upper
LC.BDNF.L	Equal variances assumed	.841	.411	-8.691	4	.001	-1.75390597	.2017963785	-2.31418253	-1.19362940
	Equal variances not assumed			-8.691	3.418	.002	-1.75390597	.2017963785	-2.35391984	-1.15389210

Tests of Normality

	group	Kolmogorov-Smirnov[a] Statistic	df	Sig.	Shapiro-Wilk Statistic	df	Sig.
LC.BDNF.R	Sham	.188	3	.	.998	3	.912
	CIBP	.281	3	.	.936	3	.513

a. Lilliefors Significance Correction

T-Test

Group Statistics

	group	N	Mean	Std. Deviation	Std. Error Mean
LC.BDNF.R	Sham	3	1.000000000	.1331764216	.0768894429
	CIBP	3	2.510454069	.3896226608	.2249487481

Independent Samples Test

		Levene's Test for Equality of Variances		t-test for Equality of Means						
									95% Confidence Interval of the Difference	
		F	Sig.	t	df	Sig. (2-tailed)	Mean Difference	Std. Error Difference	Lower	Upper
LC.BDNF.R	Equal variances assumed	3.719	.126	-6.354	4	.003	-1.51045407	.2377265776	-2.17048886	-.8504192765
	Equal variances not assumed			-6.354	2.461	.014	-1.51045407	.2377265776	-2.36994732	-.6509608160

Fig.4C

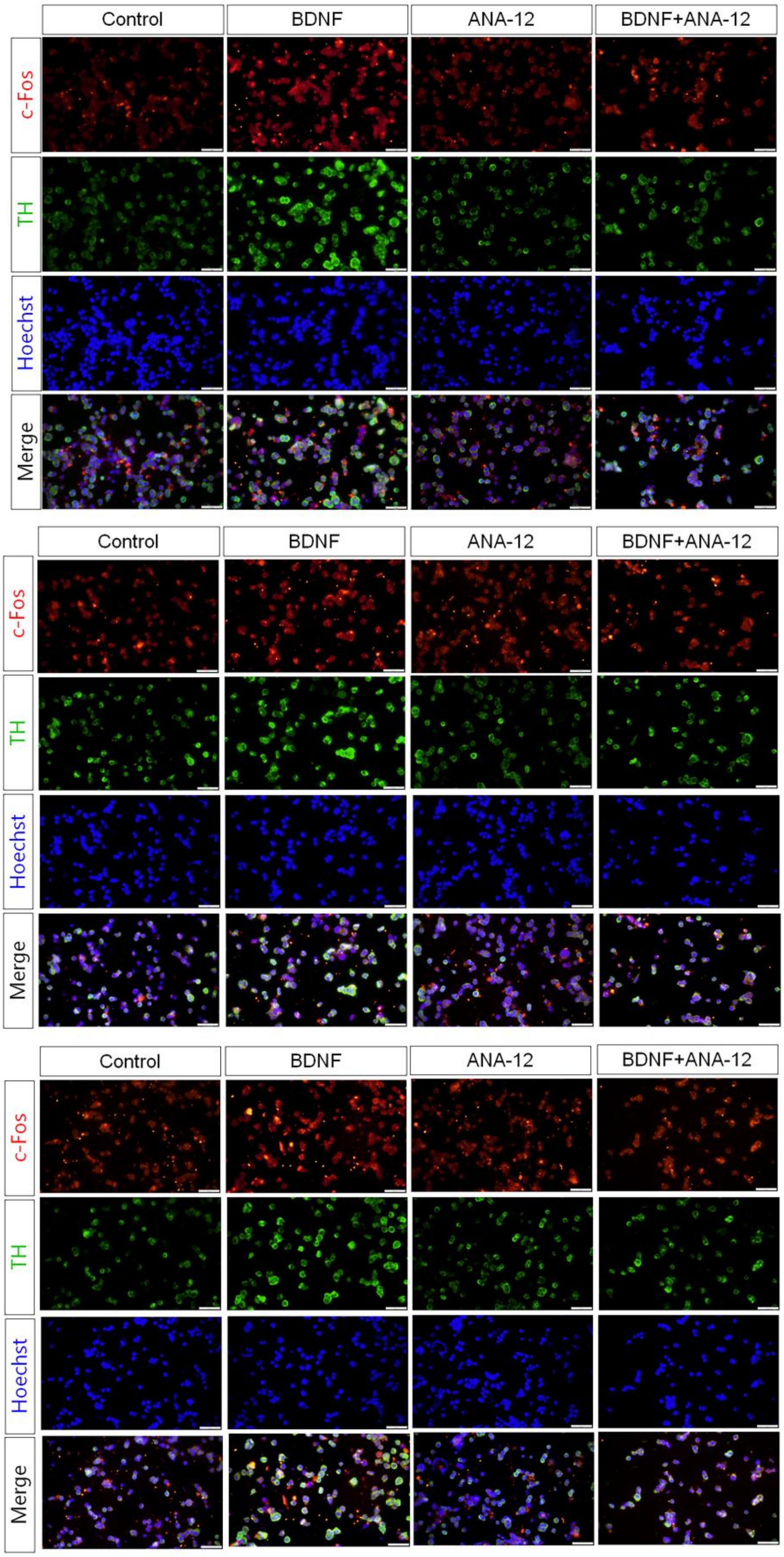
Control
BDNF
ANA-12
BDNF+ANA-12
c-Fos
TH
Hoechst
Merge
Control
BDNF
ANA-12
BDNF+ANA-12
c-Fos
TH
Hoechst
Merge
Control
BDNF
ANA-12
BDNF+ANA-12
c-Fos
TH
Hoechst
Merge

Fig.4D

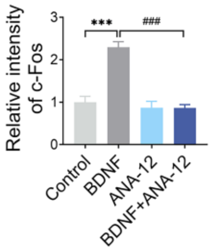

Tests of Normality

	group	Kolmogorov-Smirnov[a] Statistic	df	Sig.	Shapiro-Wilk Statistic	df	Sig.
cell.cFos	Control	.288	3	.	.929	3	.484
	BDNF	.190	3	.	.997	3	.902
	ANA-12	.234	3	.	.979	3	.720
	BDNF+ANA-12	.239	3	.	.975	3	.699

a. Lilliefors Significance Correction

Test of Homogeneity of Variances

		Levene Statistic	df1	df2	Sig.
cell.cFos	Based on Mean	.419	3	8	.744
	Based on Median	.185	3	8	.903
	Based on Median and with adjusted df	.185	3	6.773	.903
	Based on trimmed mean	.400	3	8	.757

Multiple Comparisons

Dependent Variable: cell.cFos

	(I) group	(J) group	Mean Difference (I-J)	Std. Error	Sig.	95% Confidence Interval Lower Bound	Upper Bound
Tukey HSD	Control	BDNF	-1.29380945*	.1051203733	.000	-1.630441888	-.957177012
		ANA-12	.1298464387	.1051203733	.624	-.206785999	.466478877
		BDNF+ANA-12	.1356168300	.1051203733	.593	-.201015608	.472249268
	BDNF	Control	1.29380945*	.1051203733	.000	.957177012	1.630441888
		ANA-12	1.42365589*	.1051203733	.000	1.087023451	1.760288327
		BDNF+ANA-12	1.42942628*	.1051203733	.000	1.092793842	1.766058718
	ANA-12	Control	-.1298464387	.1051203733	.624	-.466478877	.206785999
		BDNF	-1.42365589*	.1051203733	.000	-1.760288327	-1.087023451
		BDNF+ANA-12	.0057703913	.1051203733	1.000	-.330862047	.342402829
	BDNF+ANA-12	Control	-.1356168300	.1051203733	.593	-.472249268	.201015608
		BDNF	-1.42942628*	.1051203733	.000	-1.766058718	-1.092793842
		ANA-12	-.0057703913	.1051203733	1.000	-.342402829	.330862047

Fig.4E

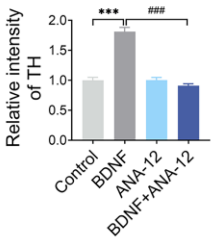

Tests of Normality

	group	Kolmogorov-Smirnov[a] Statistic	df	Sig.	Shapiro-Wilk Statistic	df	Sig.
cell.TH	Control	.179	3	.	.999	3	.948
	BDNF	.212	3	.	.990	3	.811
	ANA-12	.269	3	.	.950	3	.567
	BDNF+ANA-12	.235	3	.	.978	3	.714

a. Lilliefors Significance Correction

Test of Homogeneity of Variances

		Levene Statistic	df1	df2	Sig.
cell.TH	Based on Mean	.495	3	8	.696
	Based on Median	.324	3	8	.808
	Based on Median and with adjusted df	.324	3	6.486	.809
	Based on trimmed mean	.484	3	8	.703

Multiple Comparisons

Dependent Variable: cell.TH

	(I) group	(J) group	Mean Difference (I-J)	Std. Error	Sig.	95% Confidence Interval Lower Bound	Upper Bound
Tukey HSD	Control	BDNF	-.812056424*	.0425145567	.000	-.948203000	-.675909848
		ANA-12	-.0033571750	.0425145567	1.000	-.139503751	.132789401
		BDNF+ANA-12	.0886809743	.0425145567	.236	-.047465602	.224827550
	BDNF	Control	.8120564237*	.0425145567	.000	.675909848	.948203000
		ANA-12	.8086992487*	.0425145567	.000	.672552673	.944845825
		BDNF+ANA-12	.9007373980*	.0425145567	.000	.764590822	1.036883974
	ANA-12	Control	.0033571750	.0425145567	1.000	-.132789401	.139503751
		BDNF	-.808699249*	.0425145567	.000	-.944845825	-.672552673
		BDNF+ANA-12	.0920381493	.0425145567	.213	-.044108427	.228184725
	BDNF+ANA-12	Control	-.0886809743	.0425145567	.236	-.224827550	.047465602
		BDNF	-.900737398*	.0425145567	.000	-1.036883974	-.764590822
		ANA-12	-.0920381493	.0425145567	.213	-.228184725	.044108427

Fig.4F

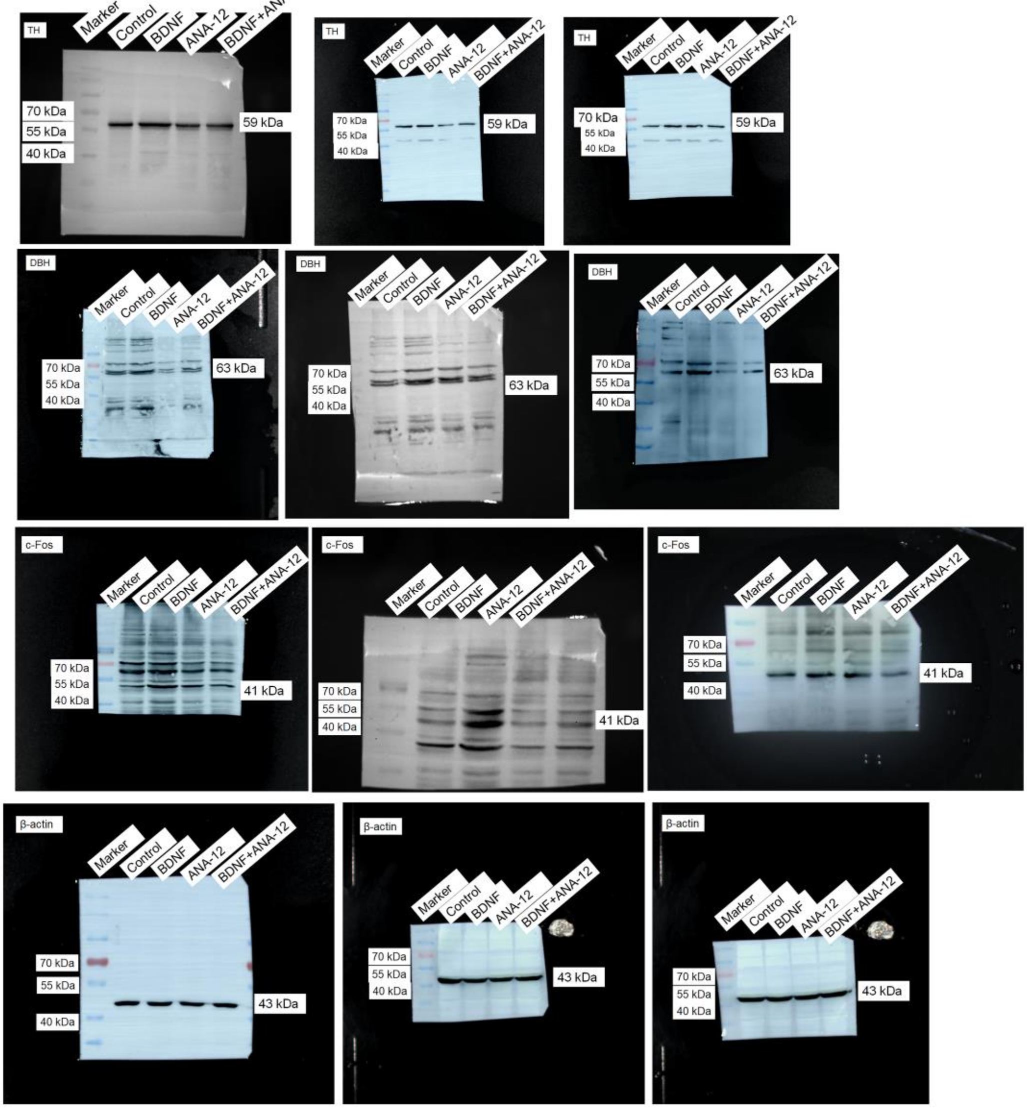
TH
Marker
Control
BDNF
ANA-12
BDNF+ANA-12
70 kDa
55 kDa
40 kDa
59 kDa
DBH
63 kDa
c-Fos
41 kDa
β-actin
43 kDa

Fig.4G

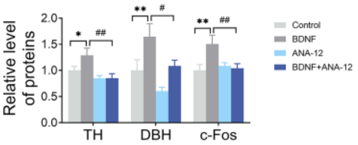

Tests of Normality

	group	Kolmogorov-Smirnov[a] Statistic	df	Sig.	Shapiro-Wilk Statistic	df	Sig.
cell.WB.TH	Control	.267	3	.	.952	3	.576
	BDNF	.175	3	.	1.000	3	.991
	ANA-12	.370	3	.	.785	3	.080
	BDNF+ANA-12	.238	3	.	.976	3	.703

a. Lilliefors Significance Correction

Test of Homogeneity of Variances

		Levene Statistic	df1	df2	Sig.
cell.WB.TH	Based on Mean	.577	3	8	.646
	Based on Median	.517	3	8	.682
	Based on Median and with adjusted df	.517	3	6.782	.684
	Based on trimmed mean	.577	3	8	.646

Multiple Comparisons

Dependent Variable: cell.WB.TH

	(I) group	(J) group	Mean Difference (I-J)	Std. Error	Sig.	95% Confidence Interval Lower Bound	Upper Bound
Tukey HSD	Control	BDNF	-.287956727*	.0766746705	.023	-.533496012	-.042417442
		ANA-12	.1517260357	.0766746705	.271	-.093813249	.397265321
		BDNF+ANA-12	.1477543957	.0766746705	.290	-.097784889	.393293681
	BDNF	Control	.2879567270*	.0766746705	.023	.042417442	.533496012
		ANA-12	.4396827627*	.0766746705	.002	.194143478	.685222048
		BDNF+ANA-12	.4357111227*	.0766746705	.002	.190171838	.681250408
	ANA-12	Control	-.1517260357	.0766746705	.271	-.397265321	.093813249
		BDNF	-.439682763*	.0766746705	.002	-.685222048	-.194143478
		BDNF+ANA-12	-.0039716400	.0766746705	1.000	-.249510925	.241567645
	BDNF+ANA-12	Control	-.1477543957	.0766746705	.290	-.393293681	.097784889
		BDNF	-.435711123*	.0766746705	.002	-.681250408	-.190171838
		ANA-12	.0039716400	.0766746705	1.000	-.241567645	.249510925

Tests of Normality

	group	Kolmogorov-Smirnov[a] Statistic	df	Sig.	Shapiro-Wilk Statistic	df	Sig.
cell.WB.DBH	Control	.219	3	.	.987	3	.782
	BDNF	.283	3	.	.934	3	.503
	ANA-12	.233	3	.	.979	3	.721
	BDNF+ANA-12	.318	3	.	.886	3	.343

a. Lilliefors Significance Correction

Test of Homogeneity of Variances

		Levene Statistic	df1	df2	Sig.
cell.WB.DBH	Based on Mean	1.761	3	8	.232
	Based on Median	.590	3	8	.638
	Based on Median and with adjusted df	.590	3	4.980	.648
	Based on trimmed mean	1.653	3	8	.253

Multiple Comparisons

Dependent Variable: cell.WB.DBH

	(I) group	(J) group	Mean Difference (I-J)	Std. Error	Sig.	95% Confidence Interval Lower Bound	Upper Bound
Tukey HSD	Control	BDNF	-.645259185*	.1431516148	.009	-1.103681045	-.186837324
		ANA-12	.3961173080	.1431516148	.092	-.062304553	.854539169
		BDNF+ANA-12	-.0855643120	.1431516148	.930	-.543986173	.372857549
	BDNF	Control	.6452591847*	.1431516148	.009	.186837324	1.103681045
		ANA-12	1.04137649*	.1431516148	.000	.582954632	1.499798353
		BDNF+ANA-12	.5596948727*	.1431516148	.019	.101273012	1.018116733
	ANA-12	Control	-.3961173080	.1431516148	.092	-.854539169	.062304553
		BDNF	-1.04137649*	.1431516148	.000	-1.499798353	-.582954632
		BDNF+ANA-12	-.481681620*	.1431516148	.040	-.940103481	-.023259759
	BDNF+ANA-12	Control	.0855643120	.1431516148	.930	-.372857549	.543986173
		BDNF	-.559694873*	.1431516148	.019	-1.018116733	-.101273012
		ANA-12	.4816816200*	.1431516148	.040	.023259759	.940103481

Tests of Normality

	group	Kolmogorov-Smirnov[a] Statistic	df	Sig.	Shapiro-Wilk Statistic	df	Sig.
cell.WB.cFos	Control	.205	3	.	.993	3	.841
	BDNF	.203	3	.	.994	3	.850
	ANA-12	.362	3	.	.804	3	.124
	BDNF+ANA-12	.305	3	.	.907	3	.407

a. Lilliefors Significance Correction

Test of Homogeneity of Variances

		Levene Statistic	df1	df2	Sig.
cell.WB.cFos	Based on Mean	.813	3	8	.522
	Based on Median	.560	3	8	.656
	Based on Median and with adjusted df	.560	3	6.548	.659
	Based on trimmed mean	.798	3	8	.529

Multiple Comparisons

Dependent Variable: cell.WB.cFos

	(I) group	(J) group	Mean Difference (I-J)	Std. Error	Sig.	95% Confidence Interval Lower Bound	Upper Bound
Tukey HSD	Control	BDNF	-.506469898*	.0927584365	.003	-.803515063	-.209424732
		ANA-12	-.0861422043	.0927584365	.791	-.383187370	.210902961
		BDNF+ANA-12	-.0429664690	.0927584365	.965	-.340011634	.254078696
	BDNF	Control	.5064698977*	.0927584365	.003	.209424732	.803515063
		ANA-12	.4203276933*	.0927584365	.008	.123282528	.717372859
		BDNF+ANA-12	.4635034287*	.0927584365	.005	.166458263	.760548594
	ANA-12	Control	.0861422043	.0927584365	.791	-.210902961	.383187370
		BDNF	-.420327693*	.0927584365	.008	-.717372859	-.123282528
		BDNF+ANA-12	.0431757353	.0927584365	.965	-.253869430	.340220901
	BDNF+ANA-12	Control	.0429664690	.0927584365	.965	-.254078696	.340011634
		BDNF	-.463503429*	.0927584365	.005	-.760548594	-.166458263
		ANA-12	-.0431757353	.0927584365	.965	-.340220901	.253869430

Fig.5D

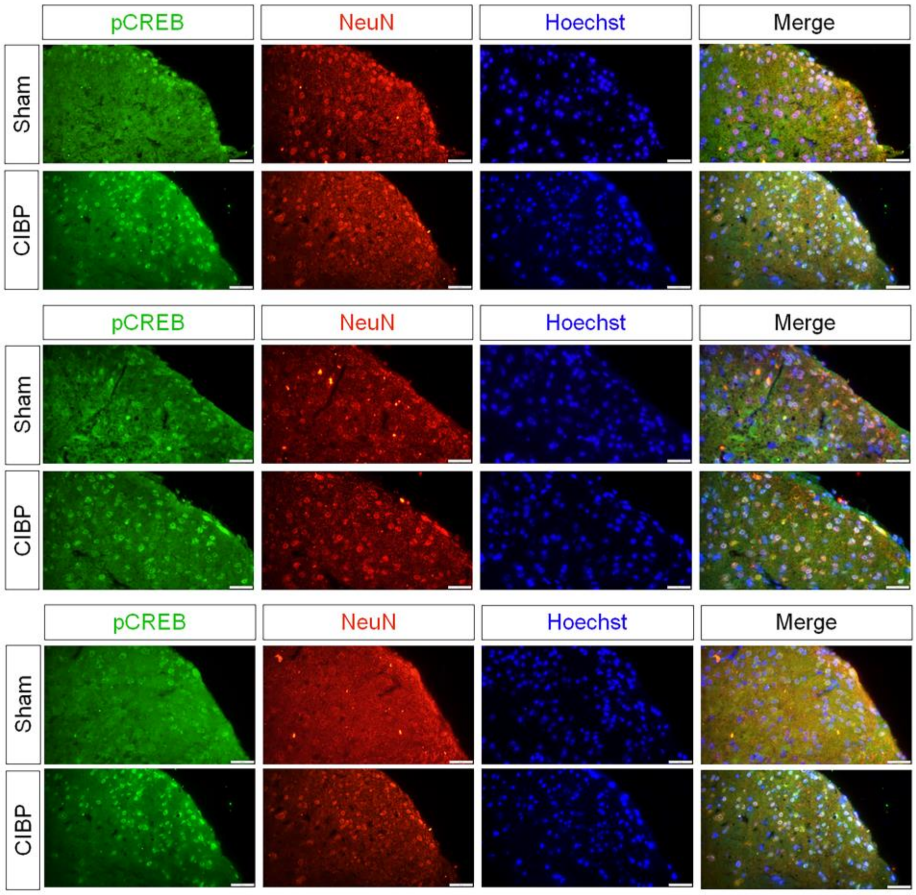

Fig.5E

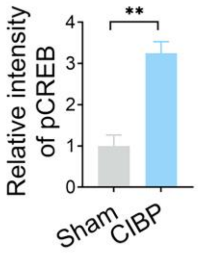

Tests of Normality

	group	Kolmogorov-Smirnov[a] Statistic	df	Sig.	Shapiro-Wilk Statistic	df	Sig.
SCpCREB	Sham	.295	3	.	.919	3	.451
	CIBP	.277	3	.	.941	3	.533

a. Lilliefors Significance Correction

T-Test

Group Statistics

	group	N	Mean	Std. Deviation	Std. Error Mean
SCpCREB	Sham	3	1.000000000	.2647826204	.1528723172
	CIBP	3	3.249035204	.2802174896	.1617836431

Independent Samples Test

		Levene's Test for Equality of Variances		t-test for Equality of Means					95% Confidence Interval of the Difference	
		F	Sig.	t	df	Sig. (2-tailed)	Mean Difference	Std. Error Difference	Lower	Upper
SCpCREB	Equal variances assumed	.010	.925	-10.104	4	.001	-2.24903520	.2225845739	-2.86702905	-1.63104135
	Equal variances not assumed			-10.104	3.987	.001	-2.24903520	.2225845739	-2.86781052	-1.63025989

Fig.5F

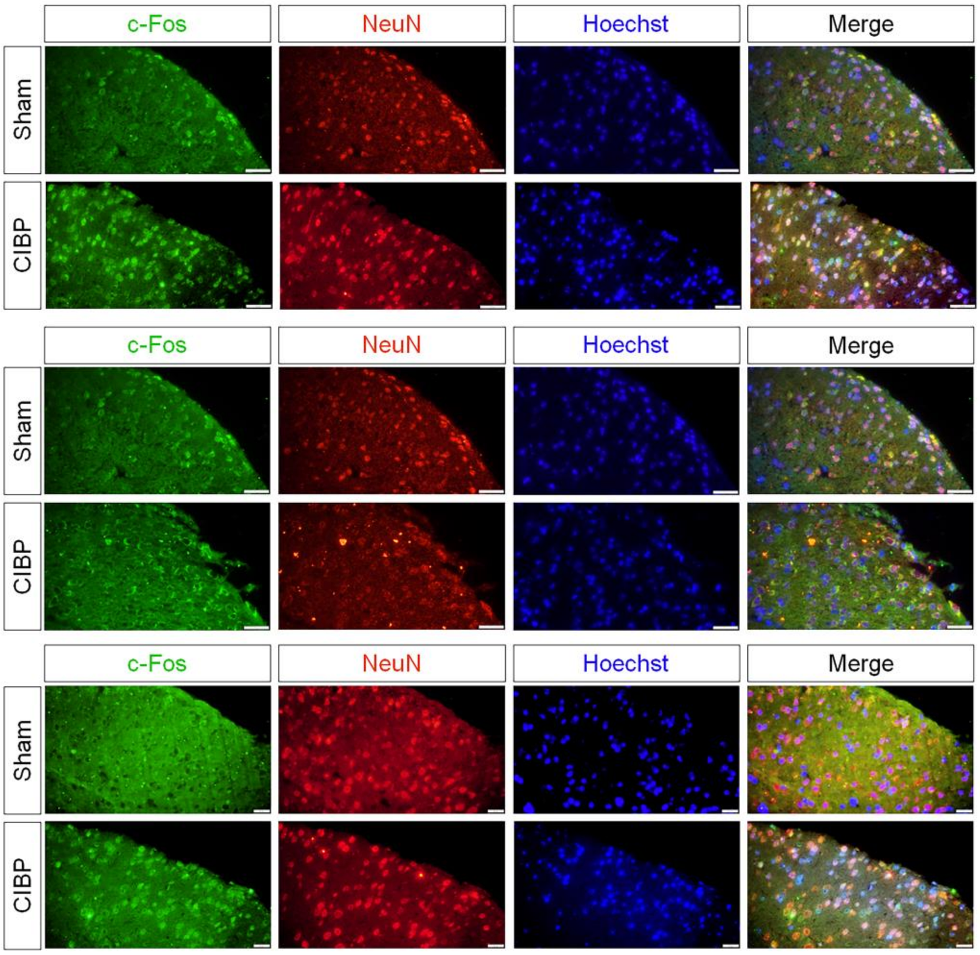

Fig.5G

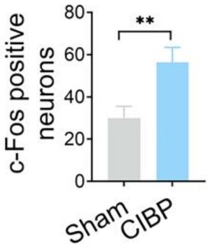

Tests of Normality

	group	Kolmogorov-Smirnov[a] Statistic	df	Sig.	Shapiro-Wilk Statistic	df	Sig.
SCcFos	Sham	.238	3	.	.976	3	.702
	CIBP	.241	3	.	.974	3	.688

a. Lilliefors Significance Correction

T-Test

Group Statistics

	group	N	Mean	Std. Deviation	Std. Error Mean
SCcFos	Sham	3	30.00000000	5.567764363	3.214550254
	CIBP	3	56.33333333	7.094598885	4.096068576

Independent Samples Test

		Levene's Test for Equality of Variances		t-test for Equality of Means					95% Confidence Interval of the Difference	
		F	Sig.	t	df	Sig. (2-tailed)	Mean Difference	Std. Error Difference	Lower	Upper
SCcFos	Equal variances assumed	.204	.675	-5.057	4	.007	-26.3333333	5.206833117	-40.7898197	-11.8768470
	Equal variances not assumed			-5.057	3.786	.008	-26.3333333	5.206833117	-41.1177358	-11.5489309

Fig.5H

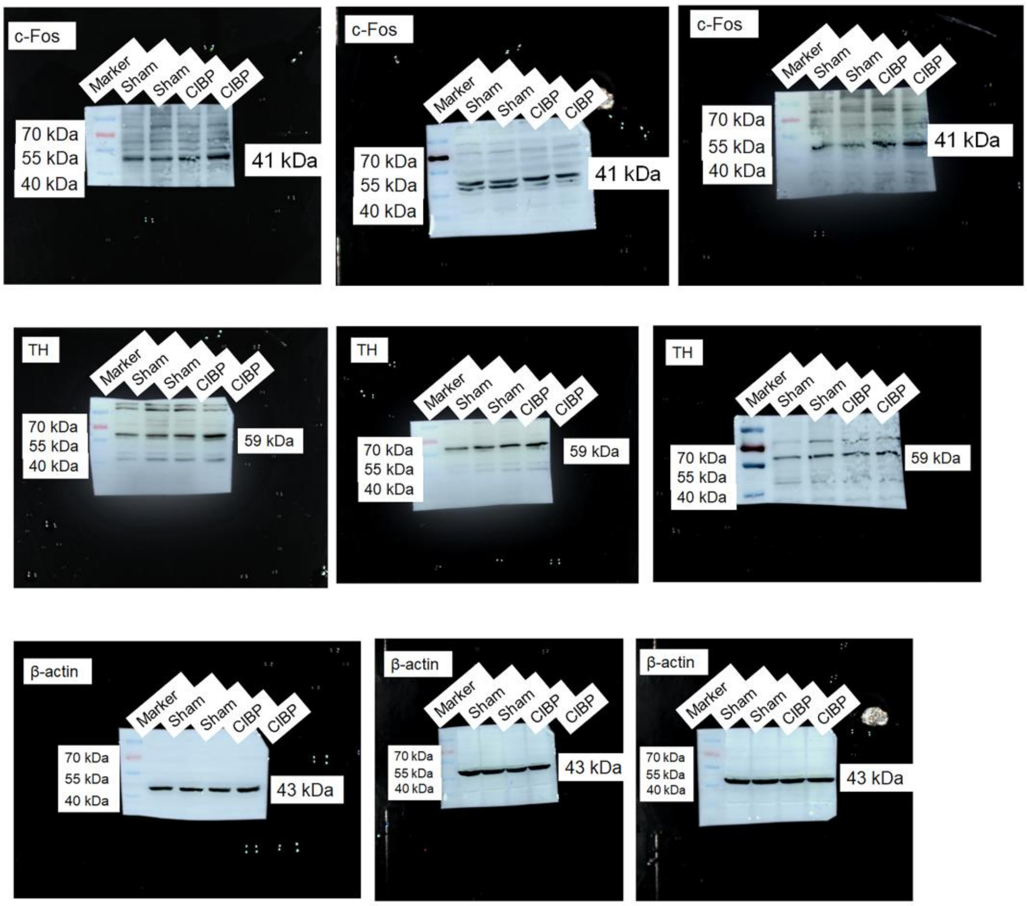
c-Fos
Marker
Sham
Sham
CIBP
CIBP
70 kDa
55 kDa
40 kDa
41 kDa
TH
59 kDa
β-actin
43 kDa

Fig.5I

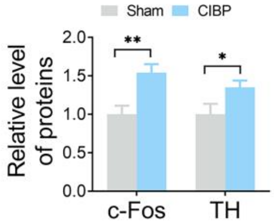

Tests of Normality

	group	Kolmogorov-Smirnov[a] Statistic	df	Sig.	Shapiro-Wilk Statistic	df	Sig.
SC.WB.cFos	Sham	.242	3	.	.973	3	.683
	CIBP	.283	3	.	.934	3	.505

a. Lilliefors Significance Correction

T-Test

Group Statistics

	group	N	Mean	Std. Deviation	Std. Error Mean
SC.WB.cFos	Sham	3	1.000000000	.1101054957	.0635694376
	CIBP	3	1.540032542	.1109489309	.0640563951

Independent Samples Test

		Levene's Test for Equality of Variances		t-test for Equality of Means						
									95% Confidence Interval of the Difference	
		F	Sig.	t	df	Sig. (2-tailed)	Mean Difference	Std. Error Difference	Lower	Upper
SC.WB.cFos	Equal variances assumed	.006	.943	-5.984	4	.004	-.5400325423	.0902457486	-.7905949094	-.2894701753
	Equal variances not assumed			-5.984	4.000	.004	-.5400325423	.0902457486	-.7906006637	-.2894644210

Tests of Normality

	group	Kolmogorov-Smirnov[a] Statistic	df	Sig.	Shapiro-Wilk Statistic	df	Sig.
SC.WB.TH	Sham	.181	3	.	.999	3	.939
	CIBP	.335	3	.	.857	3	.260

a. Lilliefors Significance Correction

T-Test

Group Statistics

	group	N	Mean	Std. Deviation	Std. Error Mean
SC.WB.TH	Sham	3	1.000000000	.1336547164	.0771655865
	CIBP	3	1.350210500	.0867724817	.0500981156

Independent Samples Test

		Levene's Test for Equality of Variances		t-test for Equality of Means						
									95% Confidence Interval of the Difference	
		F	Sig.	t	df	Sig. (2-tailed)	Mean Difference	Std. Error Difference	Lower	Upper
SC.WB.TH	Equal variances assumed	.278	.626	-3.807	4	.019	-.3502105003	.0920018963	-.6056487151	-.0947722856
	Equal variances not assumed			-3.807	3.432	.025	-.3502105003	.0920018963	-.6232305660	-.0771904346

Fig.6A

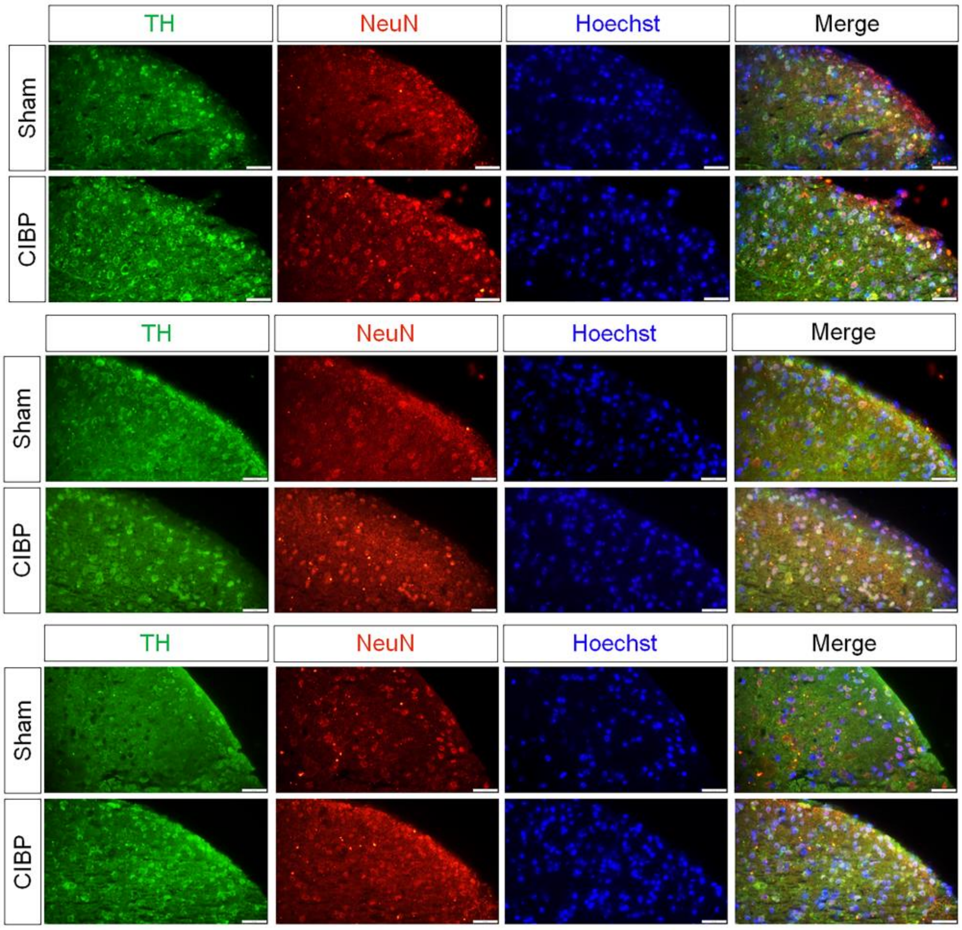

Fig.6B

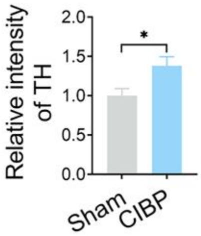

Tests of Normality

	group	Kolmogorov-Smirnov[a] Statistic	df	Sig.	Shapiro-Wilk Statistic	df	Sig.
SC.IF.TH	Sham	.230	3	.	.981	3	.734
	CIBP	.230	3	.	.981	3	.736

a. Lilliefors Significance Correction

T-Test

Group Statistics

	group	N	Mean	Std. Deviation	Std. Error Mean
SC.IF.TH	Sham	3	1.000000000	.0920534470	.0531470824
	CIBP	3	1.381662558	.1140999101	.0658756138

Independent Samples Test

		Levene's Test for Equality of Variances		t-test for Equality of Means					95% Confidence Interval of the Difference	
		F	Sig.	t	df	Sig. (2-tailed)	Mean Difference	Std. Error Difference	Lower	Upper
SC.IF.TH	Equal variances assumed	.146	.722	-4.509	4	.011	-.3816625583	.0846416497	-.6166654523	-.1466596644
	Equal variances not assumed			-4.509	3.829	.012	-.3816625583	.0846416497	-.6208684994	-.1424566172

Fig.6C

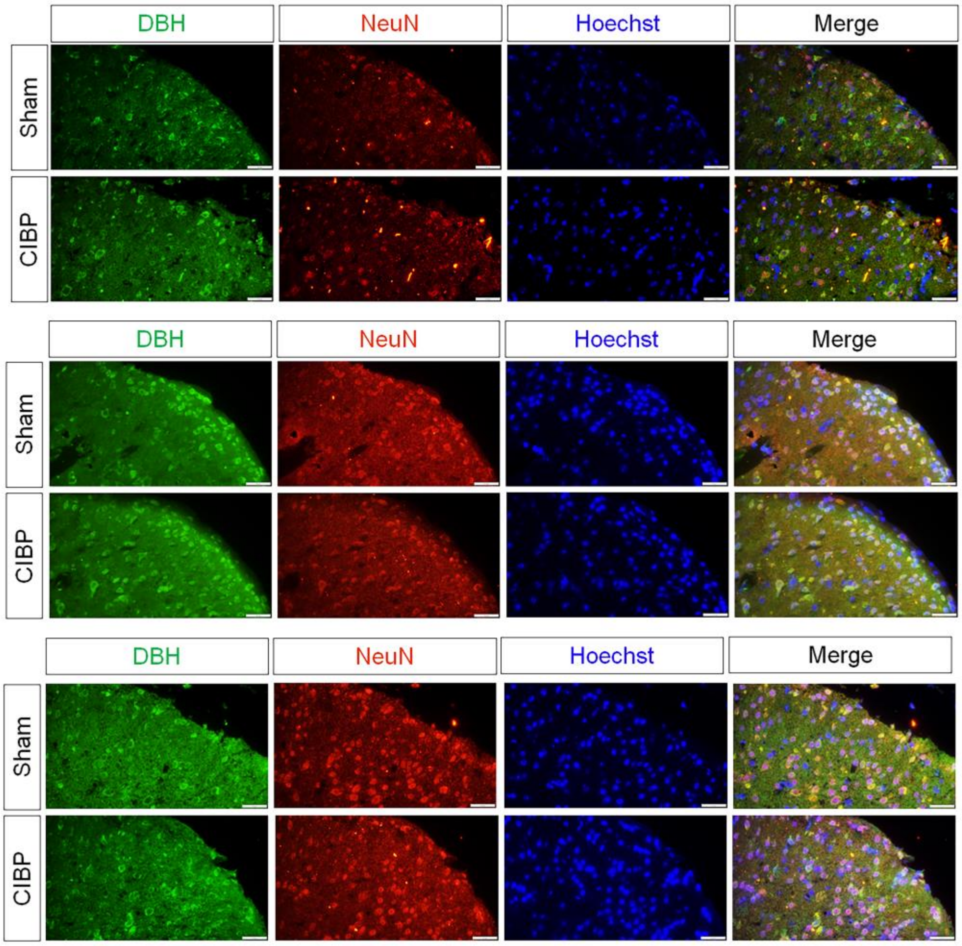

Fig.6D

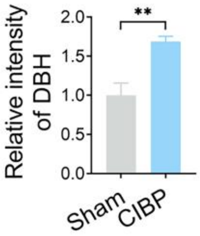

Tests of Normality

	group	Kolmogorov-Smirnov[a]			Shapiro-Wilk		
		Statistic	df	Sig.	Statistic	df	Sig.
SC.IF.DBH	Sham	.183	3	.	.999	3	.931
	CIBP	.247	3	.	.969	3	.661

a. Lilliefors Significance Correction

T-Test

Group Statistics

	group	N	Mean	Std. Deviation	Std. Error Mean
SC.IF.DBH	Sham	3	1.000000000	.1573844350	.0908659459
	CIBP	3	1.689366307	.0654824080	.0378062859

Independent Samples Test

		Levene's Test for Equality of Variances		t-test for Equality of Means						
									95% Confidence Interval of the Difference	
		F	Sig.	t	df	Sig. (2-tailed)	Mean Difference	Std. Error Difference	Lower	Upper
SC.IF.DBH	Equal variances assumed	1.254	.325	-7.005	4	.002	-.6893663070	.0984171498	-.9626161208	-.4161164932
	Equal variances not assumed			-7.005	2.672	.009	-.6893663070	.0984171498	-1.02546398	-.3532686331

Fig.6E

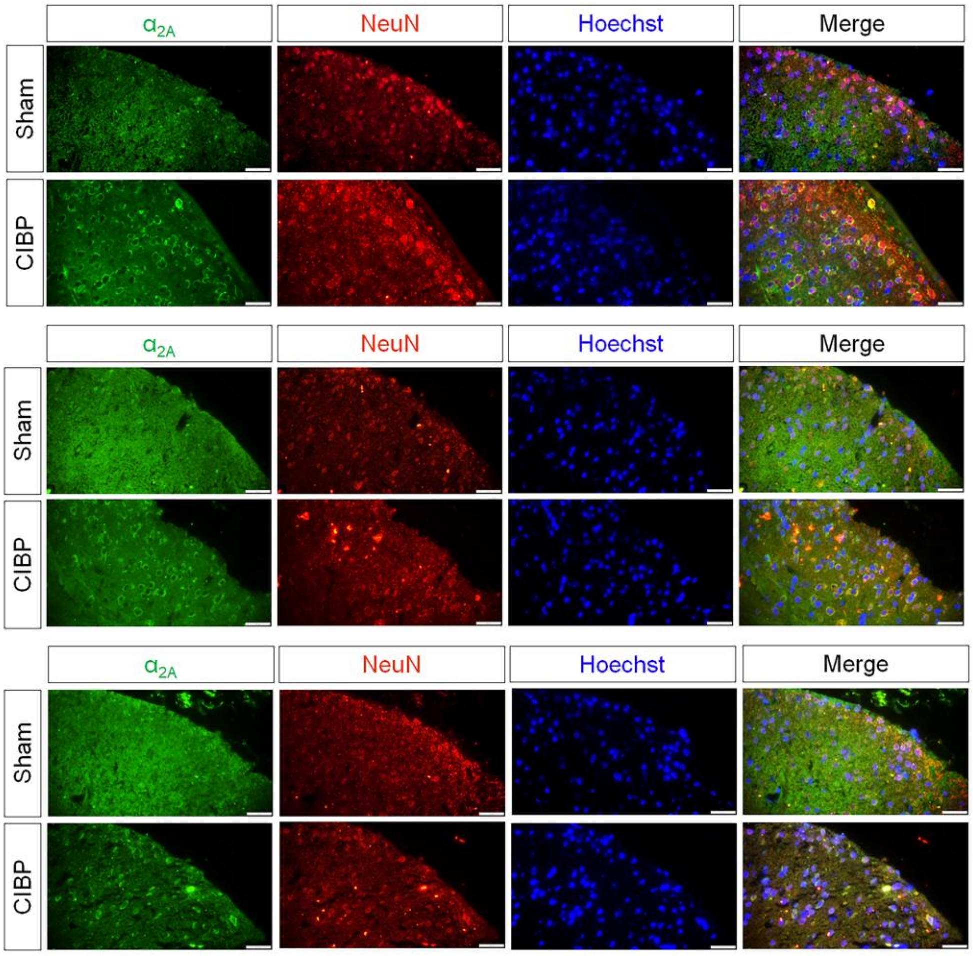

Fig.6F

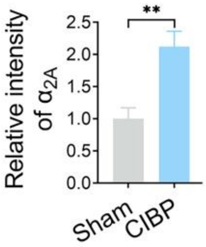

Tests of Normality

	group	Kolmogorov-Smirnov[a] Statistic	df	Sig.	Shapiro-Wilk Statistic	df	Sig.
SC.IF.α2A	Sham	.175	3	.	1.000	3	.990
	CIBP	.240	3	.	.974	3	.691

a. Lilliefors Significance Correction

T-Test

Group Statistics

	group	N	Mean	Std. Deviation	Std. Error Mean
SC.IF.α2A	Sham	3	1.000000000	.1712710637	.0988833947
	CIBP	3	2.124454880	.2361183952	.1363230190

Independent Samples Test

		Levene's Test for Equality of Variances		t-test for Equality of Means					95% Confidence Interval of the Difference	
		F	Sig.	t	df	Sig. (2-tailed)	Mean Difference	Std. Error Difference	Lower	Upper
SC.IF.α2A	Equal variances assumed	.418	.553	-6.677	4	.003	-1.12445488	.1684098907	-1.59203570	-.6568740634
	Equal variances not assumed			-6.677	3.648	.004	-1.12445488	.1684098907	-1.61036082	-.6385489418

Fig.6G

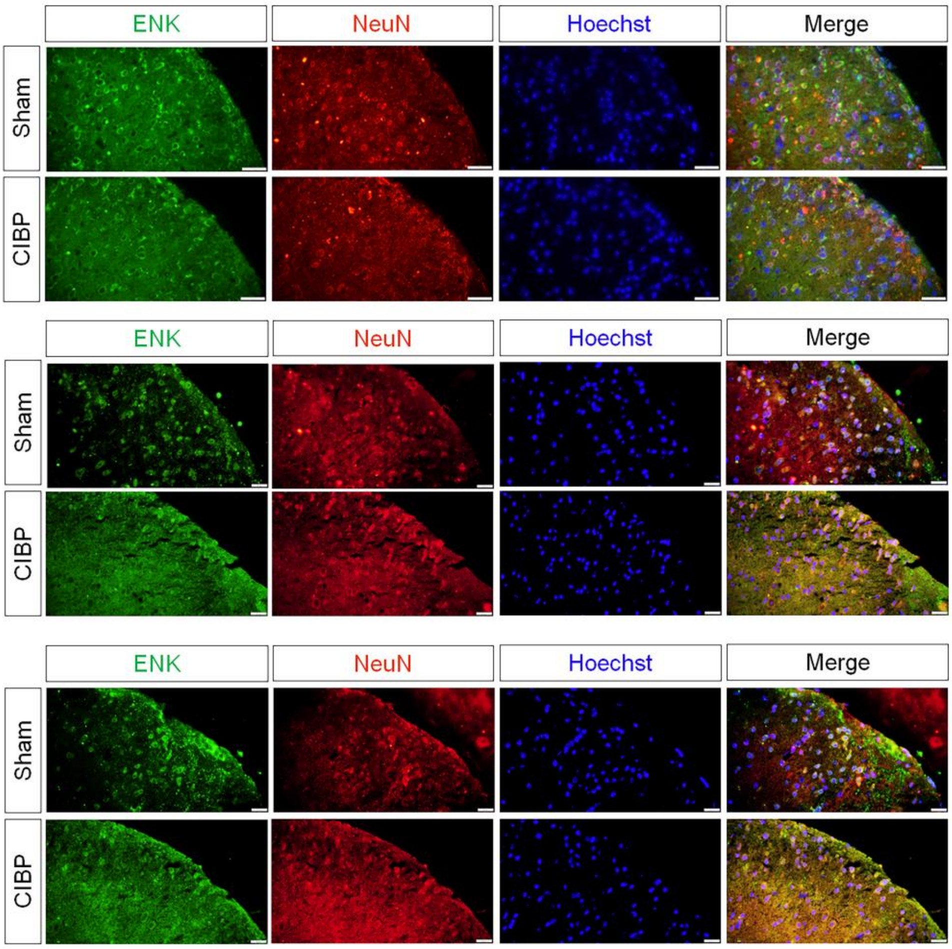

Fig.6H

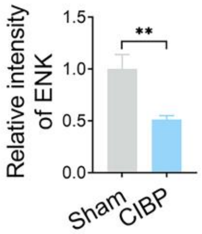

Tests of Normality

	group	Kolmogorov-Smirnov[a] Statistic	df	Sig.	Shapiro-Wilk Statistic	df	Sig.
SC.IF.ENK	Sham	.282	3	.	.936	3	.510
	CIBP	.253	3	.	.965	3	.639

a. Lilliefors Significance Correction

T-Test

Group Statistics

	group	N	Mean	Std. Deviation	Std. Error Mean
SC.IF.ENK	Sham	3	1.000000000	.1387704381	.0801191498
	CIBP	3	.513155901	.0376712436	.0217495026

Independent Samples Test

		Levene's Test for Equality of Variances		t-test for Equality of Means						
		F	Sig.	t	df	Sig. (2-tailed)	Mean Difference	Std. Error Difference	95% Confidence Interval of the Difference Lower	Upper
SC.IF.ENK	Equal variances assumed	4.732	.095	5.864	4	.004	.4868440993	.0830187872	.2563469940	.7173412046
	Equal variances not assumed			5.864	2.293	.020	.4868440993	.0830187872	.1700270050	.8036611937

Fig.6I

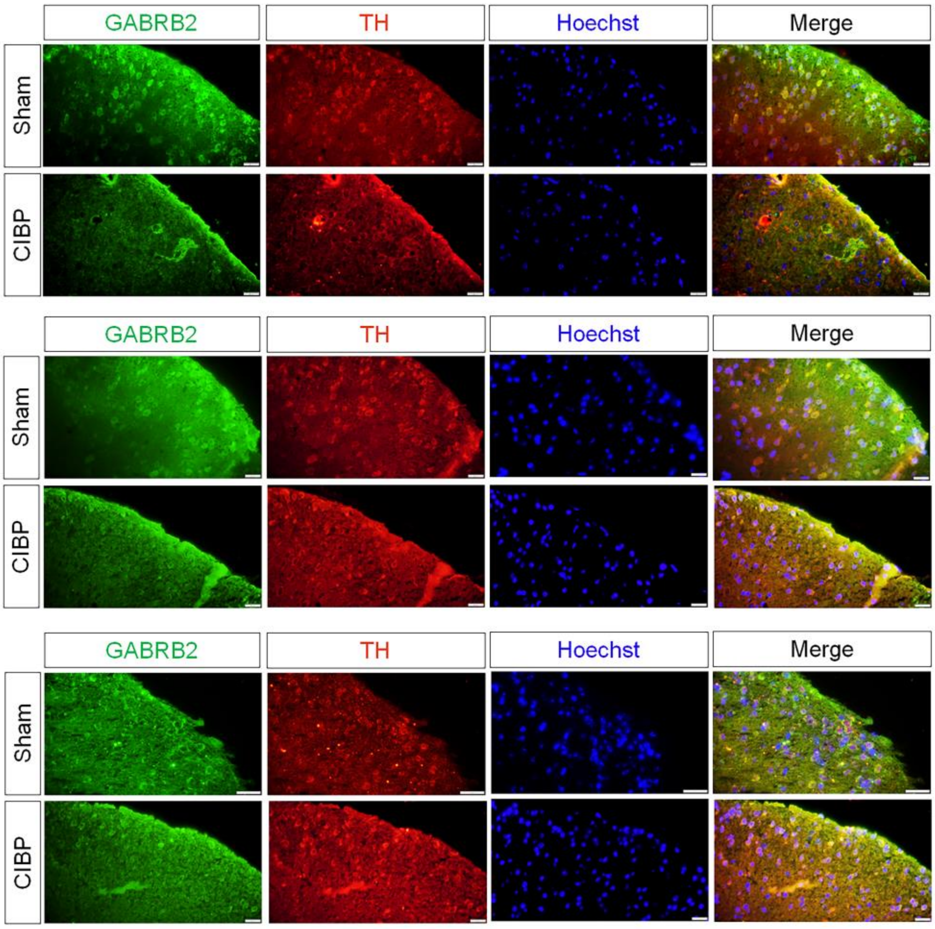

Fig.6J

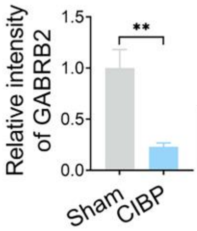

Tests of Normality

	group	Kolmogorov-Smirnov[a] Statistic	df	Sig.	Shapiro-Wilk Statistic	df	Sig.
SC.IF.GABRB2	Sham	.235	3	.	.978	3	.715
	CIBP	.218	3	.	.987	3	.784

a. Lilliefors Significance Correction

T-Test

Group Statistics

	group	N	Mean	Std. Deviation	Std. Error Mean
SC.IF.GABRB2	Sham	3	1.000000000	.1824955593	.1053638603
	CIBP	3	.230051910	.0384936819	.0222243376

Independent Samples Test

		Levene's Test for Equality of Variances		t-test for Equality of Means						
									95% Confidence Interval of the Difference	
		F	Sig.	t	df	Sig. (2-tailed)	Mean Difference	Std. Error Difference	Lower	Upper
SC.IF.GABRB2	Equal variances assumed	4.004	.116	7.150	4	.002	.7699480897	.1076822373	.4709742689	1.068921910
	Equal variances not assumed			7.150	2.178	.015	.7699480897	.1076822373	.3410233200	1.198872859
